# Neoantimycins A and B, Two Unusual Benzamido Nine-Membered Dilactones from Marine-Derived *Streptomyces antibioticus* H12-15

**DOI:** 10.3390/molecules22040557

**Published:** 2017-03-30

**Authors:** Chen Hu, Shi-Wen Zhou, Fang Chen, Xin-Heng Zheng, Hui-Fang Shen, Bi-Run Lin, Guang-Xiong Zhou

**Affiliations:** 1 College of Pharmacy, Jinan University, Guangzhou 510632, China; kitten_hc@163.com (C.H.); swqtx789@163.com (S.-W.Z.); 13751826492@163.com (F.C.); 13751866247@163.com (X.-H.Z.); 2 Institute of Plant Protection, Guangdong Academy of Agricultural Sciences, Guangzhou 510632, China; shenhf@gdppri.com (H.-F.S.); linbr@126.com (B.-R.L.)

**Keywords:** *Streptomycesantibioticus*, secondary metabolite, strain identification, neoantimycins A and B, ECD calculations, cytotoxicity, antifungal activity

## Abstract

An actinomycete strain (H12-15) isolated from a sea sediment in a mangrove district was identified as *Streptomyces* *antibioticus* on the basis of 16S rDNA gene sequence analysis as well as the investigation of its morphological, physiological, and biochemical characteristics. Two novel benzamido nonacyclic dilactones, namely neoantimycins A (**1**) and B (**2**), together with the known antimycins A_1ab_ (**3a**,**b**), A_2a_ (**4**), and A_9_ (**5**), were isolated from the culture broth of this strain. Compounds **1** and **2** are the first natural modified ATNs with an unusual benzamide unit. The structures of these new compounds, including their absolute configuration, were established on the basis of HRMS, NMR spectroscopic data, and quantum chemical ECD calculations. Their cytotoxicities against human breast adenocarcinoma cell line MCF-7, the human glioblastoma cell line SF-268, and the human lung cancer cell line NCI-H460 were also tested. All compounds exhibited mild cytotoxic activity. However, Compounds **1** and **2** showed no activity against *C. albicans* at the test concentration of 1 mg/mL via paper disc diffusion, while the known antimycins showed obvious antifungal activity.

## 1. Introduction

Marine-derived microorganisms are emerging as virtually unlimited sources for new structural classes of bioactive natural substances [1,2]. More than 50% of all known antibiotics have been originated from actinomycete bacteria, especially *Streptomyces* species [3]. To date, 46 natural antimycins A (ANTs) including deacyl antimycins have been reported [4,5,6,7,8], which exhibited antifungal [9], anticancer [10], anti-inflammatory [11], and insecticidal [12] activities. It has been proposed that ANTs are produced by a polyketide synthase (PKS)/hybrid non-ribosomal peptide synthetase (NRPS) assembly line in a biosynthetic pathway [13,14,15]. Natural ANTs possess a unique nine-membered dilactone core conjugated with a 3-formyl aminosalicylic acid (FAS) moiety [16].

During our ongoing research on bioactive secondary metabolites from *Streptomyces* [17,18,19], two novel nonacyclic dilactone fused with benzamide, neoantimycins A and B (**1**,**2**), and three known ANTs (**3**–**5**) were isolated from *Streptomyces antibioticus* H12-15 from a sea sediment collected at a mangrove district of South China Sea. Interestingly, neoantimycins A and B are the first natural modified ATNs with an unusual benzamide unit. In this paper, we describe the structure determination of two new compounds, as well as the isolation and bioactivity assay of these isolated compounds from the ethanol extract of a fermented broth of strain H12-15.

## 2. Results and Discussion

### 2.1. Characterization of the Compounds

**Figure 1:**
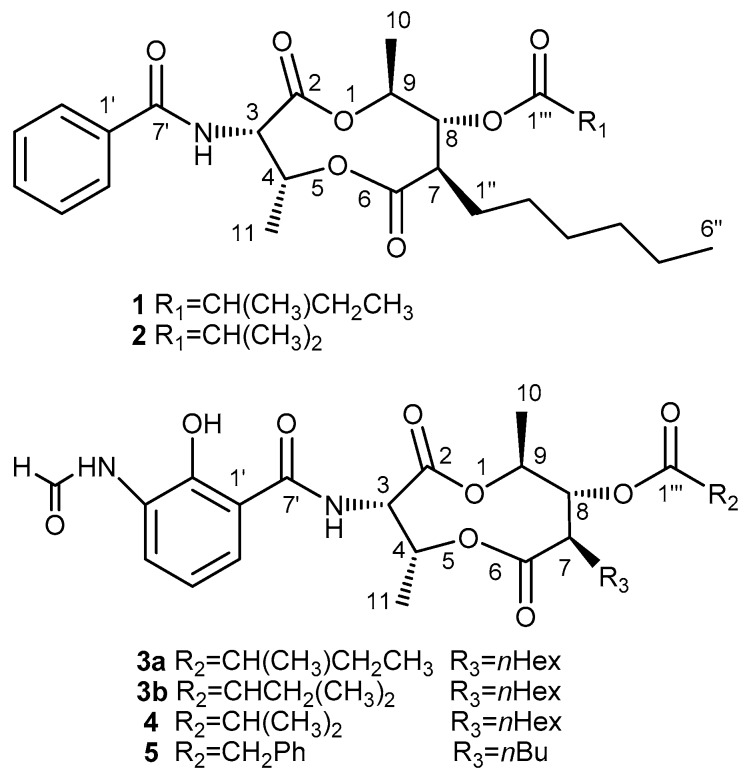
Structures of Compounds **1**–**5**.

Column chromatography and semi-preparative HPLC runs were performed on the ethyl acetate (EtOAc) extract of mycelium of *Streptomyces antibioticus* strain H12-15 to yield five compounds (**1**–**5**, Figure 1). Based on the analysis of NMR, HR-TOF-MS, and CD spectral data, as well as comparison with reported data, two of them were identified as new antimycin analogues, namely neoantimycins A (**1**) and B (**2**).

Compound **1** was obtained as a colorless crystal and yielded a molecular formula of C_27_H_39_NO_7_, with a sodium adduct at *m*/*z* 512.2621 [M + Na]^+^ detected by HRESIMS (calcd. for [C_27_H_39_NO_7_Na]^+^, 512.2625). Its ^1^H-NMR spectrum showed aromatic-ring resonances at *δ* 7.82 (2H, d, *J* = 7.0 Hz, H-2′ and 6′), 7.55 (1H, t, *J* = 7.3 Hz, H-4′), and 7.47 (2H, t, *J* = 7.6 Hz, H-3′ and 5′), suggesting the presence of a mono-substituted benzene. Four methyl doublets at *δ* 1.32 (*J* = 6.7 Hz, Me-11), 1.28 (*J* = 6.1 Hz, Me-10), 1.18 (*J* = 7.0 Hz, Me-5′′′), and 0.94 (*J* = 7.4 Hz, Me-4′′′), as well as a methyl triplet at *δ* 0.85 (*J* = 6.6 Hz, Me-6″), were also displayed in the ^1^H-NMR spectrum. There are only 25 skeleton carbons including four carbonyl carbon atoms at *δ* 175.4 (C-1′′′), 173.2 (C-6), 170.8 (C-2), and 167.0 (C-7′) and four signals of oxygenated or nitrogen-bearing carbons at *δ* 75.6 (C-8), 74.6 (C-9), 71.5 (C-4), and 54.2 (C-3) in the ^13^C-NMR spectrum, which were further confirmed by DEPT and HSQC spectra. All spectroscopic data indicated that **1** was an antimycin A_1ab_ analogue with the same dilactone macro-ring, similar to that of Compound **3**, which was also isolated from this culture broth.

**Figure 2:**
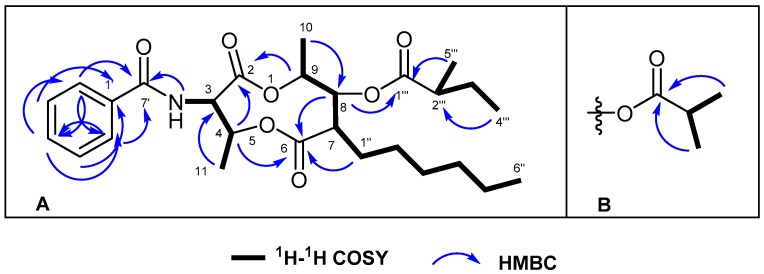
Key ^1^H–^1^H COSY and HMBC correlations for Compounds **1** (**A**) and **2** (**B**).

Comparing NMR spectroscopic data of 1 with those of **3a**, there were visible changes on the chemical shift values of those carbons at *δ* 167.0 (C-7′), 133.4 (C-1′), 132.3 (C-4′), 128.9 (C-3′ and 5′), and 127.3 (C-2′ and 6′); that is, the 1,2,3-trisubstituted benzene ring of **3a** was replaced by a symmetrical mono-substituted benzene ring. The position of this phenyl group was confirmed by 2D NMR experiments (Figure 2). The doublet of aromatic protons at *δ* 7.82 (H-2′ and 6′) displayed two HMBC correlations to C-7′ (*δ* 167.0), C-6′ (*δ* 127.3), C-4′ (*δ* 132.3), and C-2′ (*δ* 127.3), a triplet of aromatic proton at *δ* 7.55 (H-4′) showed an HMBC correlation to C-2′ and C-6′ (*δ* 127.3), other H-3′ and 5′ protons (*δ* 128.9) of the ring revealed two HMBC correlations to C-1′ (*δ* 133.4), C-3′, and C-5′ (*δ* 128.9), and a doublet at *δ* 6.36 (7′-NH) displayed an HMBC correlation to C-7′ (*δ* 167.0). In addition, ^1^H–^1^H COSY correlations from H-3 (*δ* 5.34) to H-11 (*δ* 1.32) through H-4 (*δ* 5.75) and from H-7 (*δ* 2.50) to H-10 (*δ* 1.28) and H-6″ (*δ* 0.85), as well as HMBC correlations from H-4 (*δ* 5.75) to C-3 (*δ* 54.2), C-6 (*δ* 173.2), and C-11 (*δ* 15.2), from H-7 (*δ* 2.50) to C-1″ (*δ* 133.4), C-6 (*δ* 173.2), and C-8 (*δ* 75.6) and from H-9 (*δ* 4.99) to C-2 (*δ* 170.8), C-8 (*δ* 75.6), and C-10 (δ 18.0), magnificently confirmed the structure of the dilactone ring part. Moreover, a 2-methylbutyryl fragment was identified on the basis of ^1^H–^1^H COSY correlations between H-2′′′ (*δ* 2.40), H-3′′′ (*δ* 1.75 and 1.49), H-4′′′ (*δ* 0.94), and H-5′′′ (*δ* 1.18), as well as HMBC correlations between H-2′′′ (*δ* 2.40) and C-1′′′ (*δ* 175.4). These data demonstrated that the 7-alkyl side chain is a 4-methylhexyl group, and the 8-*O*-acyl group is a 2-methylbutyryl group.

**Figure 3:**
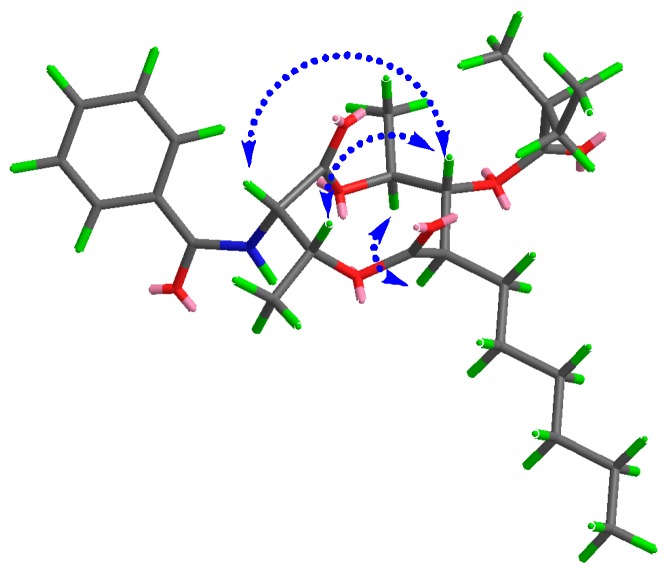
Key NOESY correlations of **1**.

**Figure 4:**
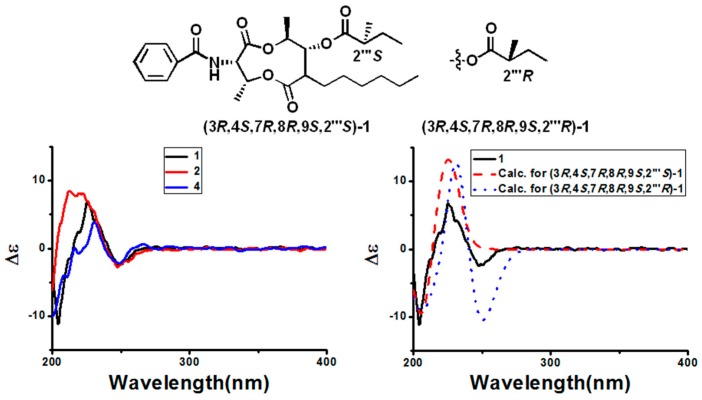
Experimental ECD spectra of **1**, **2**, **4**, and the calculated ECD spectra of (3*R*,4*S*,7*R*,8*R*,9*S*,2′′′*S*)-**1** and *(*3*R*,4*S*,7*R*,8*R*,9*S*,2′′′*R)*-**1**.

**Figure 5:**
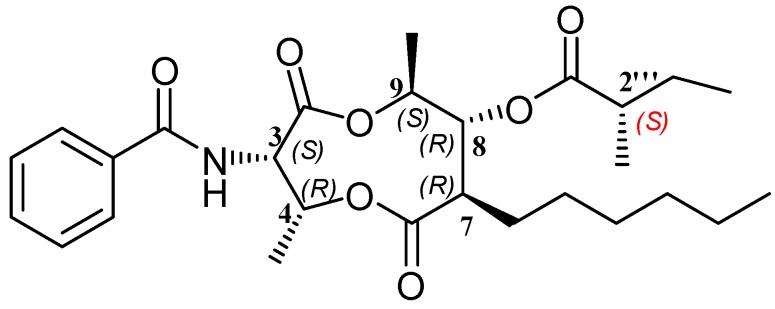
The chemical structure of **1**.

In the NOESY experiment, the visible correlations between H-3 (*δ* 5.34) and H-4 (*δ* 5.75)/H-8 (*δ* 5.09) and between H-7 (*δ* 2.50) and H-9 (*δ* 4.99) confirmed the relative configuration of **1** indicated in Figure 3. In the ECD experiment and UV spectrum, the fine structured UV band at 275–200 nm with maxima at 227 nm exhibited the same UV features for all antimycin compounds, and the CD spectra of Compounds **1** and **4** in methanol displayed similar patterns of Cotton effects (Figure 4), indicating that they possess identical basic dilactone skeletons. The absolute conformational analysis of (3*R*,4*S*,7*R*,8*R*,9*S*,2′′′*S*)-**1** and *(*3*R*,4*S*,7*R*,8*R*,9*S*,2′′′*R)*-**1** were implemented using SYBYL version 2.0 via the MMFF94 force field, which gave the lowest energy conformers. The pair of isomers were used in ECD calculations at a [PBE1PBE/6-31G] level in conjunction with the measured CD data. Unfortunately, there was no visible difference among the experimental ECD spectrum of **1** and the predicted ECD curves of both isomers (Figure 4), indicating that ECD calculations were not an adequate technique to solve the stereochemistry at C-2′′′. However, the determination of the configuration at C-2′′′ as *S* in the structurally related (+)-antimycin A_3a_ by total synthesis [20] allowed us to suggest the same *S* configuration at that chiral center of our compound on the basis of a common biosynthetic origin. According to this proposal, the structure of **1** was estabished as (3*R*,4*S*,7*R*,8*R*,9*S*, 2′′′*S*)-3-(benzoylamino)-7-hexyl-4,9-dimethyl-1,5-dioxo-2,6-dioxonan-8-yl-2′-methyl propionic ester and named as neoantimycin A (Figure 5).

Compound **2** was also isolated as a colorless crystal. Its molecular formula was established as C_26_H_37_NO_7_ on the basis of the [M + Na]^+^ ion at *m*/*z* 498.2460 ([C_26_H_37_NO_7_Na]^+^, calcd. for 498.2468) in HR-ESI-MS. The ^1^H-NMR spectrum displayed characteristic signals of ANT for aromatic-ring resonances at *δ* 7.82 (2H, d, *J* = 7.4 Hz, H-2′ and 6′), 7.55 (1H, t, *J* = 7.4 Hz, H-4′), and 7.47 (2H, t, *J* = 7.7 Hz, H-3′ and 5′), four methyl doublets at *δ* 1.32 (*J* = 6.5 Hz, Me-11), 1.28 (*J* = 6.5 Hz, Me-10), 1.22 (*J* = 2.5 Hz, Me-4′′′), and 1.21 (*J* = 2.5 Hz, Me-3′′′), and a methyl triplet at *δ* 0.86 (*J* = 6.8 Hz, Me-6″). Comparison of its ^1^H-NMR spectroscopic data of **2** with those of **1** indicated that the major difference between them was the side chain, that is, an isopropyl group with two methyl doublets at *δ* 1.22 (Me-3′′′) and 1.21 (Me-4′′′) in **2**. The ^13^C and DEPT spectra illustrated only 24 signals, including four carbonyl carbon atoms at *δ* 175.7 (C-1′′′), 173.2 (C-6), 170.8 (C-2), and 167.0 (C-7′), four signals of oxygenated or nitrogen-bearing carbons at *δ* 75.6 (C-8), 74.6 (C-9), 71.5 (C-4), and 54.2 (C-3), and two adjacent carbons [C-3′′′ (*δ* 19.1) and C-4′′′ (*δ* 19.1)]. The ^1^H-^1^H COSY correlations between H-2′′′ (*δ* 2.61) and H-3′′′ (*δ* 1.22) or H-4′′′ (*δ* 1.21), as well as the long-range HMBC correlations from the two methyl doublets to a carbonyl carbon C-1′′′ (*δ* 175.7), suggested the presence of an isobutyryl unit (Figure 2B). Interpretation of the COSY, HSQC, and HMBC data helped to construct the planar structure of **2**, and the assignments of all proton and carbon resonances are provided in Table 1.

NOE correlations between H-3 and H-4/H-8, H-7, and H-9 were observed, so the conformation of C-1~C-9 can better achieve the steric requirements. Above all, the CD spectra of Compounds **2** and **4** in methanol revealed the same Cotton effects, indicating that there is no configurational difference between these two compounds (Figure 4). Therefore, Compound **2** was established as (3*R*,4*S*,7*R*,8*R*,9*S*)-3-(benzoylamino)-7-hexyl-4,9-dimethyl-1,5-dioxo-2,6-dioxonan-8-yl-isopropyl ester and named as neoantimycin B.

In addition, three biogenetically related compounds, antimycins A_1ab_ (**3a**/**b**), A_2a_ (**4**) [16], and A_9_ (**5**) [12], were identified by HR-TOF-MS and NMR data analyses and by comparison with other data in the literature. Unfortunately, the structural similarities of the inseparable mixtures, Compounds **3a**/**b**, bearing a closely related acyl group, have made their separation via HPLC purification difficult. Based on analysis of the NMR spectra, there were two different acyl groups owing to their discrete ^1^H and ^13^C signals, although most of the ^1^H and ^13^C signals about the basic skeleton of the dilactone and benzyl rings of the two components were completely overlapped [17].

### 2.2. Biological Activity

#### 2.2.1. Antifungal Activity

The isolated metabolites **3**–**5** exhibited an obvious effect against *Candida albicans*, while the two new compounds **1**–**2** showed no bioactivity against the test strain. The antifungal activity of the extract eventually formed the known antimycins.

#### 2.2.2. Cytotoxic Assay

The cytotoxicity of Compounds **1**–**5** against the human breast adenocarcinoma cell line MCF-7, the human glioblastoma cell line SF-268, and the human lung cancer cell line NCI-H460 has been investigated by the MTT assay. *Cis*-dichlorodiamine platinum was used as the positive control. According to the result of the cytotoxicity assay (Table 2), it is worth noting that the dilactone ring may be involved directly in the interaction of antimycin A with its site of action [21]. Compounds **1** and **2** showed moderate growth inhibitory activities toward the SF-268 cell line with IC_50_ values of 33.6 μg/mL (68.7 μM) and 41.6 μg/mL (87.6 μM), respectively. The other compounds containing 3-aminosalicylic acid were much more potent against the three cell lines tested, especially SF-268 with IC_50_ values lower than 1.6 μg/mL.

## 3. Materials and Methods

### 3.1. General Materials

Melting points were recorded on an X-5 micro-MP apparatus (Huayan Corporation, Shanghai, China). The optical rotations were measured with a JASCO digital polarimeter (JASCO Corporation, Tokyo, Japan) using a thermostable optical glass cell (1 dm path length and *c* in g/100 mL). UV spectra were taken on a JASCO V-550 UV/VIS spectrometer (JASCO Corporation, Tokyo, Japan). IR spectra were carried out with a Nicolet Impact 410-FTIR instrument (Thermo, San Jose, CA, USA) in KBr pellets. All NMR spectra were obtained on Bruker AV-300 and AV-600 spectrometer (Bruker Instrument, Inc., Zurich, Switzerland) using the residual signals (CHCl_3_: *δ*_H_ 7.26/*δ*_c_ 77.5) as the internal standard. HR-ESI-MS was achieved on an Agilent 6210 LC/MS TOF mass spectrometer (Agilent Technologies, Santa Clara, CA, USA). HPLC was performed on an Agilent 1200 HPLC system equipped with a diode array detector, using a Column A (Ultimate XB-C18, 5 μm, 4.6 mm × 250 mm, Welch, Potamac, MA, USA) for analysis and a Column B (Ultimate XB-C18, 5 μm, 10 mm × 250 mm, Welch, Potamac, MA, USA) for semi-preparative purification. Open column chromatography (CC) was performed on silica gel (200–300 mesh, Haiyang Chemical Group Corporation, Qingdao, China) and Sephadex LH-20 (25–100 mm, Pharmacia, Uppsala, Sweden). HSGF254 silica gel TLC plates (0.2 mm thickness, 200 mm × 200 mm, Qingdao Marine Chemical, Qingdao, China) were used as a routine analysis of fractions. The spraying reagent used for TLC was 10% H_2_SO_4_ in EtOH. All the other reagents and solvents were purchased from Tianjin Damao Chemical Company (Tianjin, China).

### 3.2. Strain Isolation and Identification

The actinomycete strain H12-15 was isolated in 2002 from a marine sediment sample collected at the Taishan mangrove site, Guangdong province, China, and kept in a test tube with sandy soil and stored at about 4 °C in a refrigerator in the Guangdong Key Laboratory of New Technique for Plant Protection, Institute of Plant Protection, Guangdong Academy of Agricultural Sciences, Guangzhou, China, before use. Strain H12-15 was activated on a Gauserime synthetic agar medium at 28 °C. Studies on its morphological, physiological, and biochemical characteristics indicated that this strain belonged to the genus *Streptomyces*. It was further identified as a *S. antibioticus* strain based on analysis of its 16S rDNA gene sequence (see supplementary materials), which showed similarity with the sequence of *S. antibioticus* (GenBank accession NR043348).

### 3.3. Fermentation

Strain H12-15 was cultivated under shaking at 28 °C and 160 rpm for 48 h in five 1 L Erlenmeyer flasks, each containing 250 mL of the seed medium (composed of yeast powder 30 g, cornstarch 30 g, crude salt 2.5 g, CaCO_3_ 1.5 g, KNO_3_ 1 g, MgSO_4_ 0.6 g, K_2_HPO_4_ 0.9 g, FeSO4 0.02 g, H_2_O 1 L, pH 7.2–7.4). Then, the seed culture was transferred into a 30 L fermenter containing 20 L of a production medium (composed of yeast powder 30 g/L, cornstarch 30 g/L, crude salt 2.5 g/L, CaCO_3_ 1.5 g/L, KNO_3_ 1.0 g/L, MgSO_4_ 0.6 g/L, K_2_HPO_4_ 0.9 g/L, FeSO4 0.02 g/L, H_2_O 20 L, pH 7.2–7.4) and incubated for five days at 30 °C, and this was performed three times.

### 3.4. Extraction and Isolaion

The mycelium was percolated using 95% ethanol (EtOH) after centrifugation and the 95% EtOH crude extract was concentrated under reduced pressure. Then, five further consecutive partitions of the dried extract with EtOAc/H_2_O (3:1 (*v*/*v*)) were performed to yield 98 g of organic phase. The EtOAc extract was dissolved in petroleum ether and acetone and loaded on the silica gel column (60–100 mesh, Qingdao, China) after filtration. A stepwise gradient elution of petroleum ether–acetone (from 100:0 to 0:100 (*v*/*v*)) was used, and 10 fractions (Fr-1 to Fr-10) were grouped after TLC analysis. Fr-3 was subsequently applied to silica gel column chromatography eluting with a stepwise gradient of petroleum ether-EtOAc (30:1, 15:1, and 5:1 (*v*/*v*)) to yield four subfractions (Fr-3-1 to Fr-3-4). Fr-3-2 was further purified by reversed-phase semi-preparative HPLC eluting with 75% ACN to yield **1** (15.4 mg, t_R_ = 35.5 min) and **2** (5.1 mg, t_R_ = 26.9 min). Fr-7 was further chromatographed on silica gel column (petroleum ether–acetone = 15:1 to 0:1 (*v*/*v*)) and purified by semi-preparative reversed phase HPLC using 60% ACN with 0.1% TFA (trifluoroacetic acid) to obtain Compounds **3** (6.3 mg, t_R_ = 80.0 min), **4** (11 mg, *t*_R_ = 60.1 min), and **5** (6.1mg, *t*_R_ = 55.3 min). All of the above preparative HPLC separations were performed at a flow rate of 3 ml/min and detection of UV absorption at 210 nm.

Neoantimycin A (**1**): Colorless needle crystal; [α]${}_{D}^{28}$ = +11.1° (*c* 0.01, MeOH); UV (MeOH) λ_max_ (log ε): 202.8 (4.01), 227.2 (4.08) nm; IR (KBr) v_max_: 3449, 2970, 2361, 1744, 1643, 1531, 1381, 1199, 1141 cm^−1^; CD (MeOH) λ_max_ (Δε): 247.4 (−1.10), 225.4 (+3.04), 204.4 (−5.01) nm; ^1^H-NMR (CDCl_3_, 300 MHz) and ^13^C-NMR (CDCl_3_, 75 MHz), see Table 1; HRESI-TOF-MS (positive): *m*/*z* 512.2621 [M + Na]^+^ (C_27_H_39_NNaO_7_, calcd. 512.2625).

Neoantimycin B (**2**): Colorless needle crystal; [α]${}_{D}^{28}$ = +17.7° (*c* 0.01, MeOH); UV (MeOH) λ_max_ (log ε): 201.8 (3.98), 227.4 (3.97) nm; IR (KBr) v_max_: 3443, 2921, 2353, 1736, 1643, 1523, 1384, 1193, 1147, 597 cm^−1^; CD (MeOH) λ_max_ (Δε): 247.4 (−1.27), 219.6 (+3.77), 212.6 (+3.92) nm; ^1^H-NMR (CDCl_3_, 600 MHz) and ^13^C-NMR (CDCl_3_, 150 MHz), see Table 1; HRESI-TOF-MS (positive): *m*/*z* 498.2460 [M + Na]^+^ (C_26_H_37_NNaO_7_, calcd. 498.2468).

### 3.5. Computational Details for ECD of Compound ***1***

The initial 3D structures of (3*R*,4*S*,7*R*,8*R*,9*S*,2′′′*S*)-**1** and (3*R*,4*S*,7*R*,8*R*,9*S*,2′′′*R*)-**1** were generated with Chem3D version 15.0. For each molecule, the initial 3D structure was minimized with Gasteiger–Huckel force field implemented in SYBYL version 8.0 with default parameters before its conformation space was sampled. Conformer databases were also generated using SYBYL version 8.0, with an energy window for acceptable conformers (ewindow) of 5 kcal mol^−1^ above the ground state using the modified version of the MMFF94 force field mentioned above, an RMSD cutoff (rmsd) of 0.1 Å. The low energy conformers accounting for more than 5% Boltzmann distribution were further optimized successively in the gas phase by a semi-empirical method and the Hartree–Fork (HF) method at the 6-31G (d) level in Gaussian 09 program package, which was reoptimized and analyzed for frequency using the density functional theory (DFT) at the B3LYP/6-31G (d, p) level, and this was also performed in the methanol, which resulted in another epimer. Solvent effects were taken into account by using the polarizable continuum model (PCM). The conformers were calculated electronic circular dichroism (ECD) by the time-dependent density functional theory (TD-DFT) method at the PBE1PBE/6-31G (2d, p) level with the PCM model in a methanol solution. The overall calculated ECD curves were generated by the Boltzmann weighting of their selected low-energy conformers. These steps were performed with software SpecDis 1.53 with *σ* = 0.2 eV at 25 nm shift [22,23,24].

### 3.6. Biological Activities

#### 3.6.1. Antifungal Activity

Antifungal assays were performed by a paper disc diffusion assay with *Candida albicans* as an indicator. The test samples were prepared in MeOH solutions at a concentration (1 mg/mL), and the filter paper discs (6 mm) were soaked in 10 μL of the solutions. Then, the discs were placed on inoculated agar plates after evaporating the solvent and incubated in the agar Martin medium (composed of agar 18 g, glucose 20 g, peptone 5 g, yeast extract 4 g, K_2_HPO_4_·7H_2_O 0.63 g, MgSO_4_·7H_2_O 1.8 g, H_2_O 1 L) for 24 h at 37 °C.

#### 3.6.2. Cytotoxic Assay

Three cancer cell lines, MCF-7, SF-268, and NCI-H460, were obtained from School of Medicine, Jinan University (Guangzhou, China), and were cultured in RPMI 1640 medium (Life Technologies, Grand Island, NY, USA) supplemented with 10% fetal bovine serum (FBS, Gibco, Carlesbad, CA, USA), 100 U/mL penicillin and 100 μg/mL streptomycin (Invitrogen, Carlsbad, CA, USA). Cells were cultured at 37 °C in a humidified atmosphere of 5% CO_2_. The cytotoxicity of isolated compounds against three cancer cell lines was evaluated with an MTT assay with *cis*-dichlorodiamine platinum as a positive control. Seven concentrations (1.563, 3.125, 6.25, 12.5, 25, 50 and 100 μg/mL) of Compounds **1**–**5** were set for the test. Briefly, the cells were seeded at 8 × 10^4^ C/mL in 96-well plates overnight. When cells fusion reached 80% of the bottom, five compounds and *cis*-dichlorodiamine platinum at different concentrations were added and cultured for 48 h. Subsequently, MTT dye (dissolved in deionized water) was added to the 96-well plates. Following this, the 96-well plates were incubated at 37 °C for another 4 h. The liquid supernatant was removed softly and DMSO was added (100 μL per well). The absorbance at 570 nm was monitored using a microplate reader (Bio-Rad, Hercules, CA, USA). The concentrations required to inhibit cell growth by 50% (IC_50_) were calculated using Origin 8 software (OriginLab, Northampton, MA, USA).

## 4. Conclusions

Two new modified ATNs along with three known ones (**3**–**5**) were isolated from the culture broth of *Sterptomyces* sp. H12-15, which was isolated from a mangrove district and identified as *Streptomyces antibioticus* on the basis of 16S rDNA gene sequence analysis. Absolute structures of neoantimycins A (**1**) and B (**2**) were elucidated on the basis of extensive analysis of spectroscopic data, especially quantum chemical ECD calculations. The cytotoxicities of all compounds were tested by the MTT method. Neoantimycins A (**1**) and B (**2**) indicated moderate cytotoxic activity against human glioblastoma cell line SF-268 with IC_50_ values of 33.6 μg/mL (68.7 μM) and 41.6 μg/mL (87.6 μM).

## Figures and Tables

**Table 1:** ^1^H- and ^13^C-NMR spectral data of new Compounds **1** and **2** (CDCl_3_, *δ* in ppm).

No.	1		2	
	*δ*_C_^a^	*δ*_H_ (*J* in Hz) ^b^	*δ*_C_^c^	*δ*_H_ (*J* in Hz) ^d^
2	170.8	-	170.8	-
3	54.2	5.34, t, 7.8	54.2	5.34, t, 7.8
4	71.5	5.75, p, 6.7	71.5	5.75, p, 6.8
6	173.2	-	173.2	-
7	50.3	2.50, m	50.3	2.52, m
8	75.6	5.09, t, 9.9	75.6	5.08, t, 9.9
9	74.6	4.99, m	74.6	5.00, m
10	18.0	1.28, d, 6.1	18.0	1.28, d, 6.5
11	15.2	1.32, d, 6.7	15.2	1.32, d, 6.7
1′	133.4	-	133.4	-
2′	127.3	7.82, d, 7.0	127.3	7.82, d, 7.4
3′	128.9	7.47, t, 7.6	128.9	7.47, t, 7.7
4′	132.3	7.55, t, 7.3	132.3	7.55, t, 7.4
5′	128.9	7.47, t, 7.6	128.9	7.47, t, 7.7
6′	127.3	7.82, d, 7.0	127.3	7.82, d, 7.4
7′	167.0	-	167.0	-
1″	28.6	1.67, m	28.5	1.66, m
		1.25, m		1.25, m
2″	22.6	1.25, m	22.6	1.25, m
3″	27.1	1.25, m	27.2	1.25, m
4″	31.6	1.25, m	31.6	1.25, m
5″	29.1	1.25, m	29.1	1.25, m
6″	14.2	0.85, t, 6.6	14.2	0.86, t, 6.8
1′′′	175.4	-	175.7	-
2′′′	41.4	2.41, dt, 10.6, 5.3	34.3	2.61, t, 7.0
3′′′	26.6	1.75, m	19.1	1.22, d, 2.5
		1.49, m		
4′′′	11.9	0.94, t, 7.4	19.1	1.21, d, 2.5
5′′′	16.9	1.18, d, 7.0		
7′-NH	-	6.86, d, 7.8	-	6.85, d, 7.9

^a^ Measured at 300 MHz. ^b^ Measured at 75 MHz. ^c^ Measured at 600 MHz. ^d^ Measured at 150 MHz.

**Table 2:** Cytotoxic activities of **1**–**5** on MCF-7, SF-268, and NCI-H460 cancer cells lines.

Compounds	MCF-7	SF-268	NCI-H460
**1**	>50	33.6	>50
**2**	>50	41.6	>50
**3**	18.1	<1.6	21.7
**4**	36.4	<1.6	43.7
**5**	26.1	<1.6	15.5
Positive control	4.0	41.0	25.1

Concentration range: 1.6–100 μg/mL. IC_50_: half maximal inhibitory concentration.

## Supplementary Materials

Supplementary materials are available online. IR, UV, CD, HR-TOF-MS, and NMR spectra of Compounds **1** and **2** as well as other supporting data.
