# Supplementary material for: Neoantimycins A and B, Two Unusual Benzamido Nine-Membered Dilactones from Marine-Derived Streptomyces antibioticus H12-15
Source: Molecules. 2017 Mar 30;22(4):557. doi: 10.3390/molecules22040557 (PMC6154602; doi:10.3390/molecules22040557)
Supplement: Supplementary file 1 [file molecules-22-00557-s001.pdf]

Supporting Information

Neoantimycins A and B, two unusual benzamido nine-membered dilactones from marine-derived *Streptomyces antibioticus* H12-15

Chen Hu [1], Shi-Wen Zhou [1], Fang Chen[1], Xin-Heng Zheng[1], Hui-Fang Shen[2], Bi-Run Lin[2] and Guang-Xiong Zhou [1,*]

[1] College of Pharmacy, Jinan University, Guangzhou 510632, China; kitten_hc@163.com (C.H.); 1240097288@qq.com (S.-W. Z.); 1415010302@qq.com (F. C.); 916648282@qq.com (X.-H. Z.)
[2] Institute of Plant Protection, Guangdong Academy of Agricultural Sciences, Guangzhou 510632, China; shenhf@gdppri.com (H.-F.S.); linbr@126.com (B.-R.L.)
***** Correspondence: guangxzh@sina.com; Tel.: +86-20-8522-1469; Fax: +86-20-8522-1559

Contents

Table S1. 1D and 2D NMR data of 1 (δ in ppm) in $CDCl_3$. ··········· 4

Table S2. 1D and 2D NMR data of 2 (δ in ppm) in $CDCl_3$. ··········· 5

Table S3. B3LYP-Calculated relative energies (kcal mol^{-1}) and conformational population (%) for the most stable conformers of (3*R*,4*S*,7*R*,8*R*,9*S*,2'''*S*)-**1**. ··········· 6

Table S4. Coordinates of computations ((3*R*,4*S*,7*R*,8*R*,9*S*,2'''*S*)-**1**) ··········· 6

Table S5. B3LYP-Calculated relative energies (kcal mol^{-1}) and conformational population (%) for the most stable conformers of (3*R*,4*S*,7*R*,8*R*,9*S*,2'''*R*)-**1**. ··········· 10

Table S6. Coordinates of computations ((3*R*,4*S*,7*R*,8*R*,9*S*,2'''*R*)-**1**) ··········· 10

Table S7. 2D Structure of compound **1**. ··········· 12

Figure S1. B3LYP/6-31 G optimized lowest energy 3D conformer of **1**. ··········· 14

Figure S2. Experimental ECD spectra of 1 and calculated ECD spectra for (3*R*,4*S*,7*R*,8*R*,9*S*,2'''*S*)-**1** and (3*R*,4*S*,7*R*,8*R*,9*S*,2'''*R*)-**1**. ··········· 14

The spectra of neoantimycin A (**1**) ··········· 15

Figure S3. IR spectrum of compound **1**. ··········· 15

Figure S4. CD spectrum and UV spectrum of compound **1** in MeOH. ··········· 16

Figure S5. HR-TOF-MS of compound **1**. ··········· 17

Figure S6. The ^{1}H-NMR spectrum of compound **1**. ··········· 17

Figure S7. The ^{1}H-NMR spectrum of compound **1**. ··········· 18

Figure S8. The ^{13}C-NMR spectrum of compound **1**. ··········· 18

Figure S9. The ^{13}C-NMR and DEPT spectra of compound **1**. ··········· 19

Figure S10. The ^{1}H-^{1}H COSY spectrum of compound **1**. ··········· 19

Figure S11. The HSQC spectrum of compound **1**. ··········· 20

Figure S12. The HMBC spectrum of compound **1**. ··········· 20

The spectra of neoantimycin B (**2**) ··········· 21

Figure S15. CD spectrum and UV spectrum of compound **2** in MeOH. ··········· 22

Figure S16. HR-TOF-MS of compound **2**. ··········· 22

Figure S17. The ^{1}H-NMR spectrum of compound **2**. ··········· 23

Figure S19. The ^{13}C-NMR spectrum of compound **2**. ··········· 24

Figure S20. The ^{13}C-NMR and DEPT spectra of compound **2**. ··········· 24

Figure S21. The ^{1}H-^{1}H COSY spectrum of compound **2**. ··········· 25

Figure S22. The HSQC spectrum of compound **2**. ············ 25

Figure S23. The HMBC spectrum of compound **2**. ············ 26

Figure S24. The NOESY spectrum of compound **2**. ············ 26

Figure S25. Phylogenetic tree of H12-15. ············ 28

Figure S26. 16S rDNA gene sequence of H12-15. ············ 29

Figure S27. Scaning electron micrographs of strain H12-15 on Gauze No.1 medium ······· 29

Table S1. 1D and 2D NMR data of **1** (δ in ppm) in $CDCl_3$.

No.	δ_C	δ_H (J in Hz)	^1H-^1H COSY	HMBC	NOE
2	170.8	-	-	-	-
3	54.2	5.34, t, 7.8	H-4, 7'-NH	C-2, 4, 11	H-4, H-8
4	71.5	5.75, p, 6.7	H-3, H-11	C-2, 3, 6, 11	H-3, H-8
6	173.2	-	-	-	-
7	50.3	2.50, m	H-8, H-1‴	C-6, 8, 1'', 2''	H-9
8	75.6	5.09, t, 9.9	H-7, H-9	C-7, 9, 10, 1'', 1'''	H-3, H-4
9	74.6	4.99, m	H-8, H-10	C-7, 8, 11	H-7
10	18.0	1.28, d, 6.1	H-9	C-2, 8, 9	-
11	15.2	1.32, d, 6.7	H-4	2-2, 3	-
1'	133.4	-	-	-	-
2'	127.3	7.82, d, 7.7	H-3'	C-1', 3', 4', 7'	-
3'	128.9	7.47, t, 7.7	H-2', H-4'	C-1', 2', 4', 5'	-
4'	132.3	7.55, t, 7.4	H-3', H-5'	C-2', 3', 5', 6'	-
5'	128.9	7.47, t, 7.7	H-4', H-6'	C-1', 3', 4', 6'	-
6'	127.3	7.82, d, 7.7	H-5'	C-1', 4', 5', 7'	-
7'	167.0	-	-	-	-
1''	28.6	1.67, m<br>1.25, m	H-7, H-2''	C-6, 8	-<br>-
2''	22.6	1.25, m	H-1'', H-3''	-	-
3''	27.1	1.25, m	H-2'', H-4''	-	-
4''	31.6	1.25, m	H-3'', H-5''	-	-
5''	29.1	1.25, m	H-4'', H-6''	-	-
6''	14.2	0.85, t, 6.6	H-5''	C-4'', 5''	-
1'''	175.4	-	-	-	-
2'''	41.4	2.41, dt, 10.6, 5.3	H-3''', H-5'''	C-1''', 3''', 4''', 5'''	-
3'''	26.6	1.71, m<br>1.49, m	H-2''', H-4'''	C-1''', 2''', 4''', 5'''	-<br>-
4'''	11.9	0.94, t, 7.4	H-3'''	C-2''', 3'''	-
5'''	16.9	1.18, d, 7.0	H-2'''	C-1''', 2''', 3'''	-
7'-NH	-	6.86, d, 7.8	H-2	C-2, 7'	-

Table S2. 1D and 2D NMR data of **2** (δ in ppm) in $CDCl_3$.

**No.**	**δc**	**δ_H (J in Hz)**	**^{1}H-^{1}H COSY**	**HMBC**	**NOE**
2	170.8	-	-	-	-
3	54.2	5.34, t, 7.8	H-4, 7'-NH	C-2, 4, 11	H-4, H-8
4	71.5	5.75, p, 6.8	H-3, H-11	C-2, 3, 6, 11	H-3, H-8
6	173.2	-	-	-	-
7	50.3	2.52, m	H-8, H-1‴	C-6, 8, 1", 2"	H-9
8	75.6	5.08, t, 9.9	H-7, H-9	C-7, 9, 10, 1", 1'''	H-3, H-4
9	74.6	5.00, m	H-8, H-10	C-7, 8, 11	H-7
10	18.0	1.28, d, 6.5	H-9	C-2, 8, 9	-
11	15.2	1.32, d, 6.7	H-4	C-2, 3	-
1'	133.4	-	-	-	-
2'	127.3	7.82, d, 7.4	H-3'	C-1', 3', 4', 7'	-
3'	128.9	7.47, t, 7.7	H-2', H-4'	C-1', 2', 4', 5'	-
4'	132.3	7.55, t, 7.4	H-3', H-5'	C-2', 3', 5', 6'	-
5'	128.9	7.47, t, 7.7	H-4', H-6'	C-1', 3', 4', 6'	-
6'	127.3	7.82, d, 7.4	H-5'	C-1', 4', 5', 7'	-
7'	167.0	-	-	-	-
1"	28.6	1.66, m<br>1.25, m	H-7, H-2"	C-6, 8	-<br>-
2"	22.6	1.25, m	H-1", H-3"	-	-
3"	27.1	1.25, m	H-2", H-4"	-	-
4"	31.6	1.25, m	H-3", H-5"	-	-
5"	29.1	1.25, m	H-4", H-6"	-	-
6"	14.2	0.86, t, 6.8	H-5"	C-4", 5"	-
1'''	175.7	-	-	-	-
2'''	34.3	2.61, t, 7.0	H-3''', H-5'''	C-1''', 3''', 4'''	-
3'''	19.1	1.22, d, 2.5	H-2''', H-4'''	C-1''', 2''', 4'''	-<br>-
4'''	19.1	1.21, d, 2.5	H-2''', H-3'''	C-2''', 3'''	-
7'-NH	-	6.86, d, 7.8	H-2	C-2, 7'	-

Table S3. B3LYP-Calculated relative energies (kcal mol^{-1}) and conformational population (%) for the most stable conformers of (3*R*,4*S*,7*R*,8*R*,9*S*,2'''*S*)-**1**.

Conformers	Rel. E	Percent (%)
**1-a**	0	58.46%
**1-b**	0.239705	39.00%
**1-c**	2.579025	0.75%
**1-d**	2.582163	0.75%
**1-e**	2.756607	0.56%
**1-f**	2.826260	0.49%
**1-g**	3.123695	0.30%
**1-h**	3.177660	0.27%
**1-i**	3.202760	0.26%
**1-j**	3.371557	0.20%
**1-k**	3.384735	0.19%
**1-l**	3.519020	0.15%
**1-m**	3.567965	0.14%
**1-n**	3.763745	0.10%
**1-o**	3.935052	0.08%

Table S4. Coordinates of computations ((3*R*,4*S*,7*R*,8*R*,9*S*,2'''*S*)-**1**)

Conformer 1-a

Standard orientation:

Center Number	Atomic Number	Atomic Type	Coordinates (Angstroms) X	Y	Z
1	6	0	-6.366453	0.356599	-0.229171
2	6	0	-7.379725	0.693531	-1.139236
3	6	0	-8.721485	0.531648	-0.795832
4	6	0	-9.068459	0.041184	0.467093
5	6	0	-8.066109	-0.284736	1.384750
6	6	0	-6.721988	-0.129672	1.038944
7	6	0	-4.946535	0.562165	-0.679845
8	7	0	-3.964676	-0.042257	0.054049
9	8	0	-4.671567	1.248826	-1.665850
10	6	0	-2.572343	0.033430	-0.337409
11	6	0	-1.849128	1.377274	0.106034
12	8	0	-0.503679	1.046848	0.550592
13	6	0	0.460416	0.856082	-0.389945

14	6	0	1.664009	0.124689	0.203588
15	6	0	1.627526	-1.370666	-0.224370
16	8	0	0.365988	1.215382	-1.542361
17	6	0	0.361662	-2.145820	0.206875
18	8	0	-0.737481	-1.438186	-0.433480
19	6	0	-1.840059	-1.152499	0.279639
20	8	0	-2.184760	-1.722162	1.295026
21	6	0	0.336632	-3.603030	-0.233804
22	8	0	2.742846	-2.018335	0.433449
23	6	0	3.641880	-2.715499	-0.320999
24	6	0	4.724487	-3.335167	0.552423
25	6	0	6.089963	-3.322094	-0.158377
26	8	0	3.552435	-2.843397	-1.523007
27	6	0	6.652362	-1.918646	-0.411713
28	6	0	4.295189	-4.770804	0.927521
29	6	0	2.970388	0.814963	-0.237727
30	6	0	3.105311	2.252443	0.286055
31	6	0	4.432976	2.912484	-0.112663
32	6	0	4.562888	4.360934	0.377414
33	6	0	5.890560	5.025619	-0.012203
34	6	0	6.005811	6.476299	0.470837
35	6	0	-2.512580	2.094122	1.269112
36	1	0	-7.092083	1.082583	-2.110697
37	1	0	-9.496909	0.790331	-1.512152
38	1	0	-10.114198	-0.081625	0.737140
39	1	0	-8.328437	-0.654885	2.372341
40	1	0	-5.964640	-0.369655	1.780817
41	1	0	-4.181996	-0.670296	0.817542
42	1	0	-2.504526	-0.030959	-1.426257
43	1	0	-1.775691	2.026800	-0.767178
44	1	0	1.579068	0.160367	1.296525
45	1	0	1.757323	-1.452875	-1.306896
46	1	0	0.227082	-2.069041	1.290149
47	1	0	0.442551	-3.684429	-1.320509
48	1	0	-0.610654	-4.060139	0.067784
49	1	0	1.148873	-4.162675	0.238744
50	1	0	4.784939	-2.742549	1.473688
51	1	0	5.998843	-3.863029	-1.108144
52	1	0	6.793265	-3.889900	0.465428
53	1	0	6.003560	-1.340259	-1.079505
54	1	0	7.640132	-1.975939	-0.883480
55	1	0	6.764462	-1.357873	0.525439
56	1	0	4.195667	-5.394360	0.031700
57	1	0	3.340744	-4.780053	1.465434

58	1	0	5.050947	-5.225344	1.577814
59	1	0	3.816162	0.220997	0.126267
60	1	0	3.017872	0.814667	-1.333854
61	1	0	2.275394	2.864223	-0.094048
62	1	0	3.015414	2.251908	1.383027
63	1	0	5.269853	2.317480	0.283773
64	1	0	4.532880	2.890126	-1.208122
65	1	0	4.453610	4.386361	1.472698
66	1	0	3.728995	4.955215	-0.026108
67	1	0	6.002795	4.994769	-1.105817
68	1	0	6.724463	4.436422	0.396928
69	1	0	5.931379	6.537948	1.564381
70	1	0	5.206168	7.098876	0.049177
71	1	0	6.964136	6.921129	0.176735
72	1	0	-2.600786	1.443056	2.145473
73	1	0	-1.908386	2.965285	1.542709
74	1	0	-3.508972	2.442327	0.985815

Conformer 1-b

Standard orientation:

Center Number	Atomic Number	Atomic Type	Coordinates (Angstroms) X	Y	Z
1	6	0	-6.332634	0.362910	-0.313724
2	6	0	-6.781908	-0.824866	0.281115
3	6	0	-8.136128	-1.000456	0.565103
4	6	0	-9.051982	0.007742	0.258702
5	6	0	-8.611827	1.188890	-0.344174
6	6	0	-7.260815	1.362820	-0.633834
7	6	0	-4.894653	0.627521	-0.655049
8	7	0	-3.961128	-0.135941	-0.009694
9	8	0	-4.561963	1.491540	-1.464828
10	6	0	-2.551019	-0.009506	-0.304815
11	6	0	-1.877928	1.310183	0.260158
12	8	0	-0.521434	0.988855	0.673125
13	6	0	0.425564	0.855908	-0.292925
14	6	0	1.661709	0.144755	0.257106
15	6	0	1.637683	-1.351369	-0.165886
16	8	0	0.295790	1.241464	-1.431820
17	6	0	0.388880	-2.139294	0.287836
18	8	0	-0.713144	-1.476740	-0.389663
19	6	0	-1.832753	-1.205628	0.305564
20	8	0	-2.194017	-1.785306	1.307201

21	6	0	0.398903	-3.613317	-0.092177
22	8	0	2.782054	-1.973324	0.461181
23	6	0	3.640793	-2.688310	-0.323152
24	6	0	4.789976	-3.246465	0.505729
25	6	0	6.064542	-3.384728	-0.343360
26	8	0	3.475290	-2.872673	-1.507925
27	6	0	6.637312	-2.049207	-0.828563
28	6	0	4.352548	-4.598593	1.108316
29	6	0	2.937389	0.854506	-0.235503
30	6	0	3.083199	2.282886	0.306938
31	6	0	4.378904	2.968255	-0.146683
32	6	0	4.526927	4.398207	0.388055
33	6	0	5.820773	5.090855	-0.059074
34	6	0	5.958468	6.518517	0.479408
35	6	0	-2.560017	1.879574	1.492120
36	1	0	-6.088173	-1.633449	0.495367
37	1	0	-8.476066	-1.927529	1.018401
38	1	0	-10.106516	-0.129939	0.482256
39	1	0	-9.323092	1.972514	-0.590544
40	1	0	-6.897937	2.266253	-1.112719
41	1	0	-4.202664	-0.715530	0.783125
42	1	0	-2.407523	-0.013650	-1.387125
43	1	0	-1.836391	2.041830	-0.547054
44	1	0	1.616229	0.179908	1.351958
45	1	0	1.739490	-1.434521	-1.251083
46	1	0	0.241517	-2.025326	1.366119
47	1	0	0.531749	-3.738773	-1.170989
48	1	0	-0.548269	-4.070251	0.208196
49	1	0	1.208479	-4.137685	0.422863
50	1	0	4.969793	-2.546522	1.331082
51	1	0	5.842689	-4.026064	-1.204293
52	1	0	6.815753	-3.909768	0.260917
53	1	0	5.927507	-1.526889	-1.478883
54	1	0	7.557048	-2.205543	-1.402745
55	1	0	6.880167	-1.388627	0.013274
56	1	0	4.122047	-5.320568	0.316869
57	1	0	3.467848	-4.485597	1.743558
58	1	0	5.159231	-5.012958	1.722276
59	1	0	3.806628	0.262202	0.071059
60	1	0	2.924087	0.874970	-1.331947
61	1	0	2.226024	2.888674	-0.017269
62	1	0	3.050011	2.260821	1.406433
63	1	0	5.241944	2.367128	0.176587
64	1	0	4.414050	2.985193	-1.245691

65	1	0	4.486814	4.381506	1.487665
66	1	0	3.664006	4.997965	0.062551
67	1	0	5.860516	5.108437	-1.157457
68	1	0	6.683350	4.491733	0.266230
69	1	0	5.957636	6.530585	1.576454
70	1	0	5.128060	7.150953	0.142005
71	1	0	6.890822	6.985581	0.142114
72	1	0	-2.639636	1.129155	2.285659
73	1	0	-1.972195	2.721762	1.869586
74	1	0	-3.559199	2.242340	1.241516

Table S5. B3LYP-Calculated relative energies (kcal mol^{-1}) and conformational population (%) for the most stable conformers of (3*R*,4*S*,7*R*,8*R*,9*S*,2'''*R*)-**1**.

Conformers	Rel. E	Percent (%)
**1-a**	0	81.17
**1-b**	1.714957	4.48
**1-c**	1.736292	4.32
**1-d**	1.787747	3.96
**1-e**	1.910110	3.22
**1-f**	1.986665	2.83
**1-g**	1.988547	2.82
**1-h**	2.262137	1.78
**1-i**	2.423405	1.35
**1-j**	2.956152	0.55

Table S6. Coordinates of computations ((3*R*,4*S*,7*R*,8*R*,9*S*,2'''*R*)-**1**)

Conformer 2-a

Standard orientation:

Center	Atomic	Atomic	Coordinates (Angstroms)		
Number	Number	Type	X	Y	Z
1	6	0	-6.146977	-0.128755	-0.310721
2	6	0	-7.160333	0.800115	-0.583149
3	6	0	-8.494287	0.477633	-0.346802
4	6	0	-8.831407	-0.782142	0.154678
5	6	0	-7.829282	-1.719272	0.413043
6	6	0	-6.492486	-1.395497	0.181791
7	6	0	-4.734579	0.297492	-0.590297
8	7	0	-3.744004	-0.429124	0.011583
9	8	0	-4.471475	1.255606	-1.315166

10	6	0	-2.347304	-0.145348	-0.233631
11	6	0	-1.815572	1.171510	0.470002
12	8	0	-0.426302	0.960964	0.845573
13	6	0	0.512391	1.011564	-0.136172
14	6	0	1.830985	0.401166	0.340309
15	6	0	1.946327	-1.068072	-0.157711
16	8	0	0.318762	1.470805	-1.238226
17	6	0	0.783098	-1.997800	0.252108
18	8	0	-0.377005	-1.420158	-0.405069
19	6	0	-1.526286	-1.316338	0.287111
20	8	0	-1.840005	-2.007383	1.232669
21	6	0	0.950589	-3.445014	-0.188632
22	8	0	3.140514	-1.624135	0.438384
23	6	0	4.099277	-2.136046	-0.388426
24	6	0	5.301965	-2.621641	0.409495
25	6	0	5.930083	-3.863669	-0.245847
26	8	0	4.004033	-2.164585	-1.594650
27	6	0	5.019531	-5.095510	-0.251269
28	6	0	6.318541	-1.465461	0.521356
29	6	0	3.010577	1.257273	-0.157737
30	6	0	3.054580	2.659712	0.464993
31	6	0	4.240355	3.491278	-0.044040
32	6	0	4.389681	4.867493	0.628278
33	6	0	3.223182	5.849633	0.418336
34	6	0	2.982446	6.242759	-1.043993
35	6	0	-2.540949	1.523031	1.757224
36	1	0	-6.875710	1.767625	-0.983256
37	1	0	-9.272282	1.206822	-0.555751
38	1	0	-9.872335	-1.035766	0.336680
39	1	0	-8.087925	-2.706000	0.787190
40	1	0	-5.729261	-2.149196	0.356283
41	1	0	-3.943605	-1.095415	0.745754
42	1	0	-2.184227	-0.033065	-1.307109
43	1	0	-1.865760	1.985347	-0.253822
44	1	0	1.819217	0.377445	1.436374
45	1	0	2.052606	-1.084325	-1.245516
46	1	0	0.620334	-1.946849	1.333031
47	1	0	0.057151	-4.012833	0.085625
48	1	0	1.811226	-3.899323	0.310057
49	1	0	1.096124	-3.509804	-1.271624
50	1	0	4.951530	-2.872388	1.418068
51	1	0	6.857861	-4.094600	0.293429
52	1	0	6.213501	-3.608980	-1.273721
53	1	0	4.112587	-4.915152	-0.838707

54	1	0	4.718442	-5.375476	0.766124
55	1	0	5.531406	-5.956991	-0.693834
56	1	0	6.660447	-1.153809	-0.471617
57	1	0	5.885097	-0.596039	1.026562
58	1	0	7.191639	-1.790777	1.097155
59	1	0	3.945735	0.734408	0.070728
60	1	0	2.942400	1.339536	-1.249020
61	1	0	2.115504	3.183339	0.245659
62	1	0	3.114417	2.572111	1.560423
63	1	0	5.167937	2.924314	0.120463
64	1	0	4.153720	3.613605	-1.132255
65	1	0	5.313326	5.336229	0.259566
66	1	0	4.534916	4.718404	1.708102
67	1	0	3.431118	6.756306	1.002473
68	1	0	2.300269	5.429043	0.839724
69	1	0	3.889838	6.665252	-1.494031
70	1	0	2.190476	6.996153	-1.122984
71	1	0	2.678569	5.384934	-1.654255
72	1	0	-2.049230	2.381474	2.224795
73	1	0	-3.578646	1.792575	1.548927
74	1	0	-2.523419	0.686251	2.463336

Table S7. 2D Structure of compound **1**.

Label	Structure
**1**	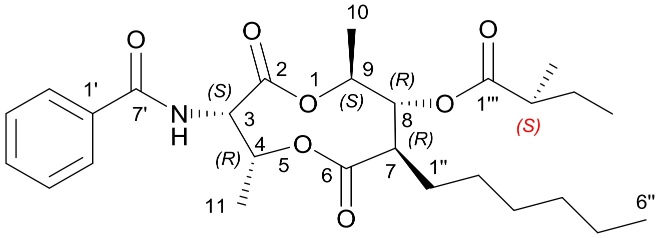

Label	Conformer	Boltzmann weighting factors
**1-a**	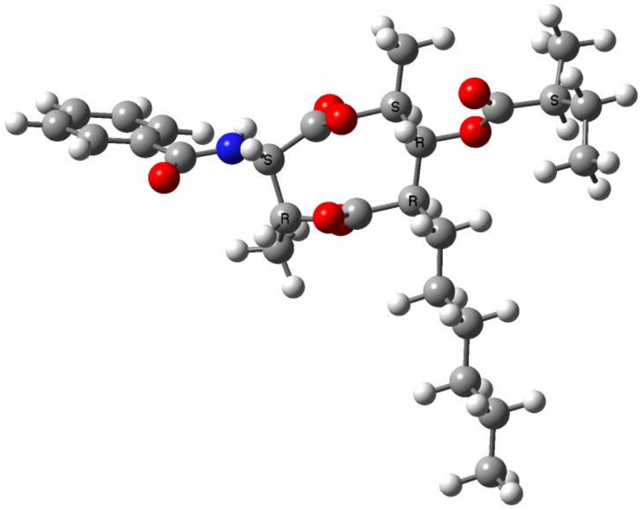	**58.46%**

**1-b**	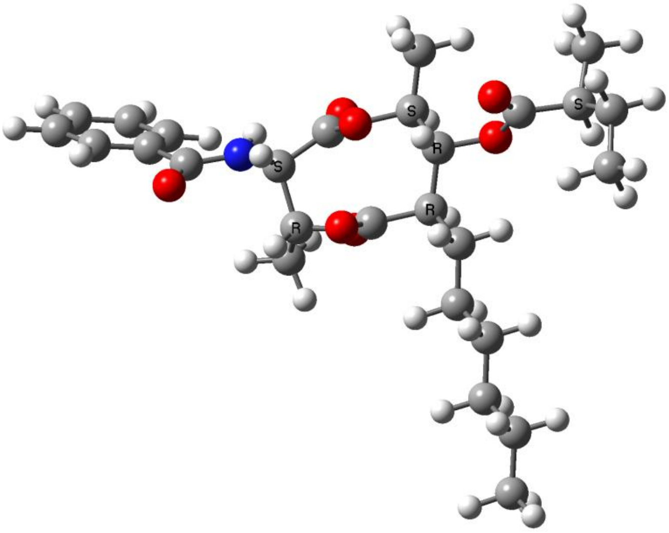	**39.00%**

Figure S1. B3LYP/6-31 G optimized lowest energy 3D conformer of **1**.

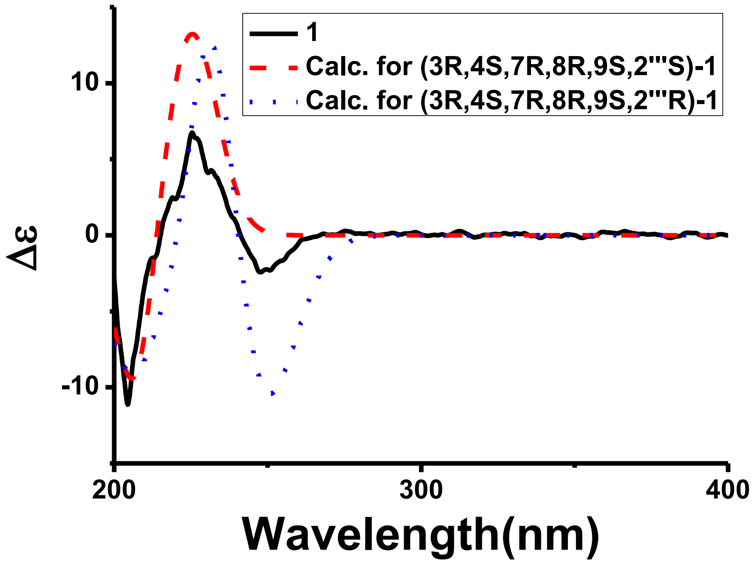

Figure S2. Experimental ECD spectra of **1** and calculated ECD spectra for (3*R*,4*S*,7*R*,8*R*,9*S*,2'''*S*)-**1** and (3*R*,4*S*,7*R*,8*R*,9*S*,2'''*R*)-**1**.

The spectra of neoantimycin A (1)

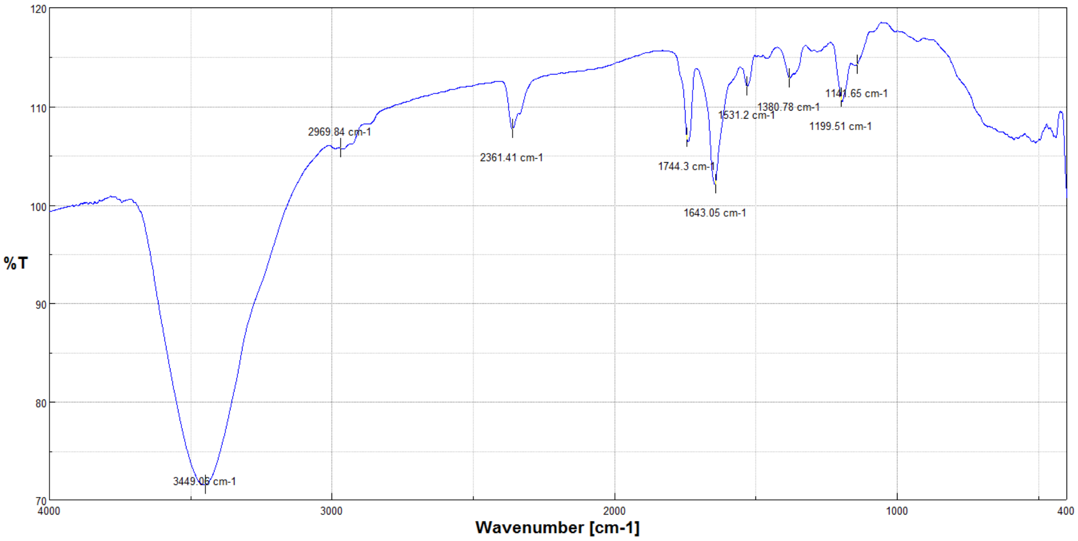

Figure S3. IR spectrum of compound 1.

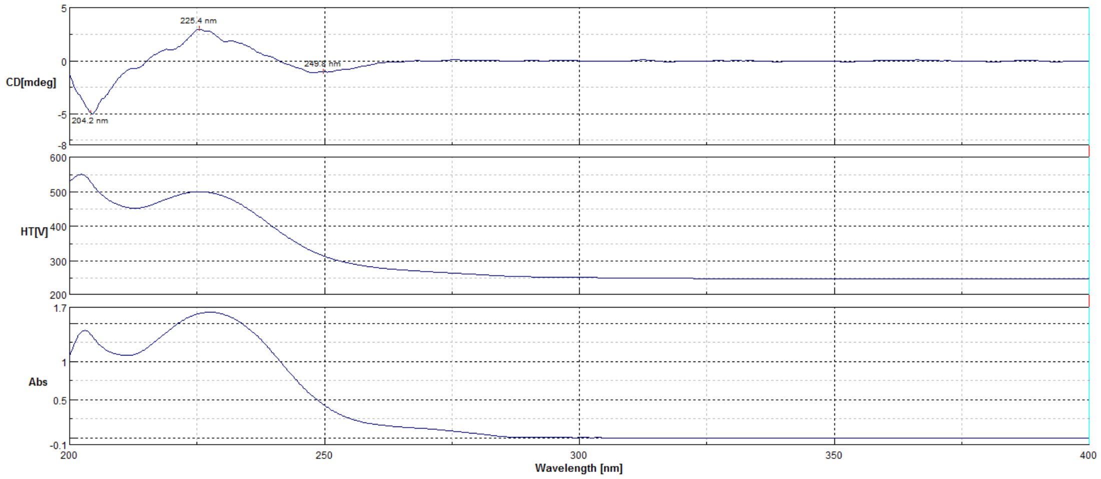

Figure S4. CD spectrum and UV spectrum of compound **1** in MeOH.

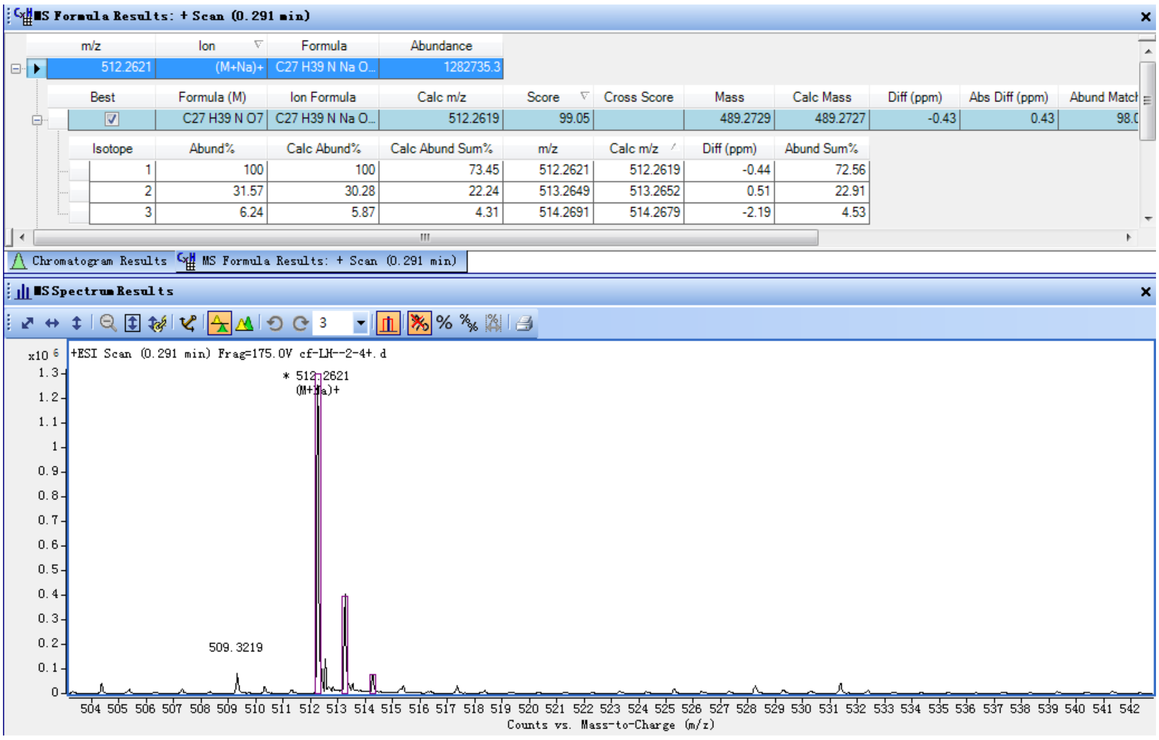

Figure S5. HR-TOF-MS of compound **1**.

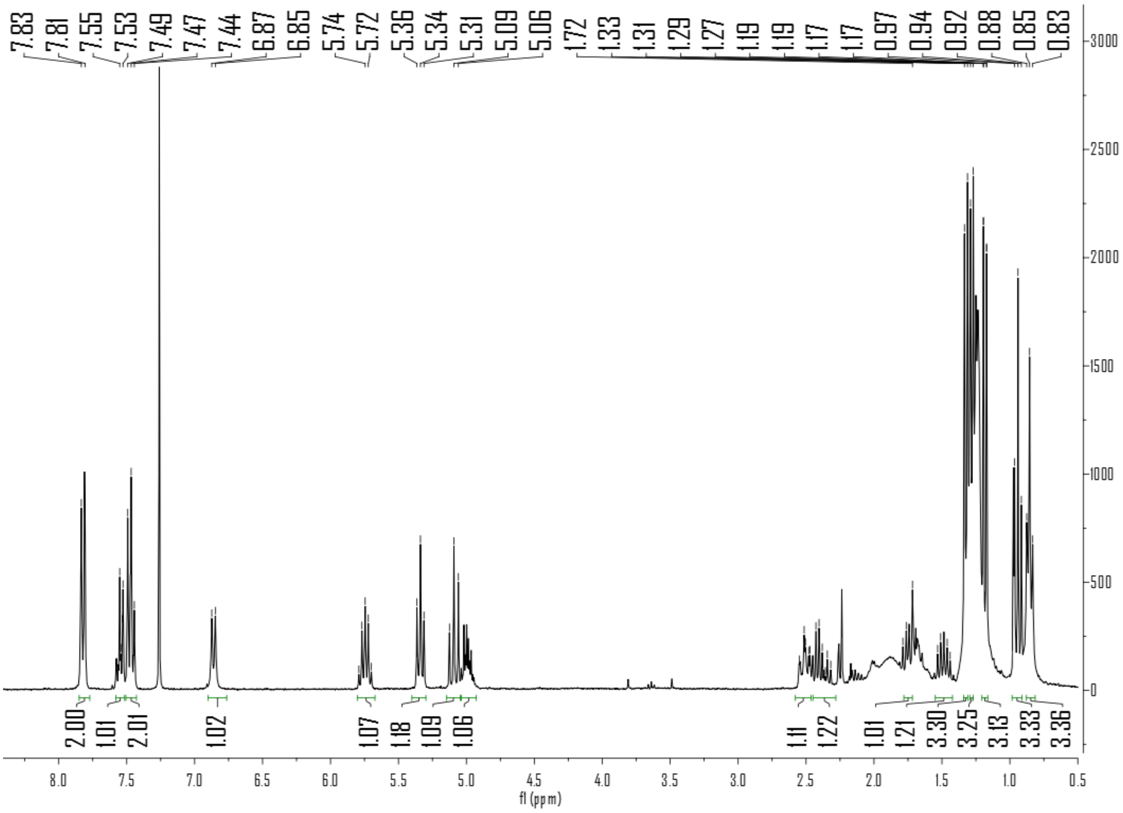

Figure S6. The ^{1}H-NMR spectrum of compound **1**.

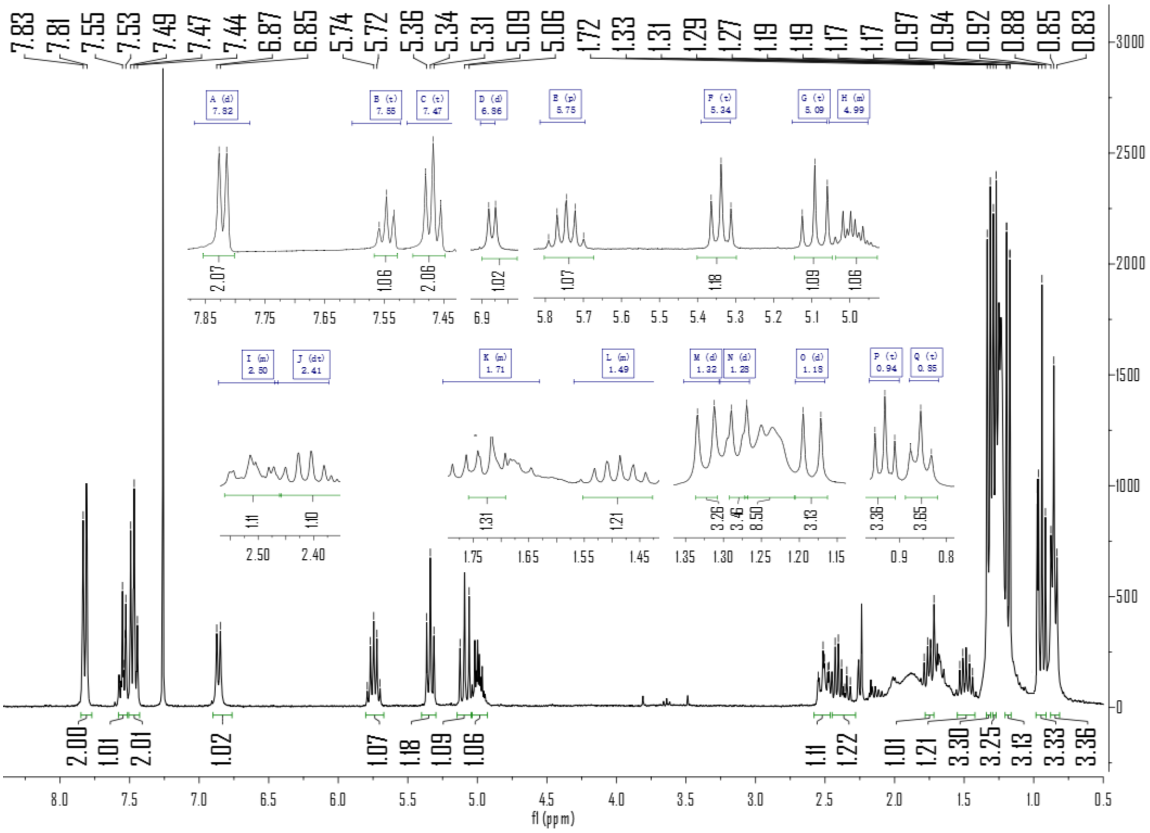

Figure S7. The ^{1}H-NMR spectrum of compound **1**.

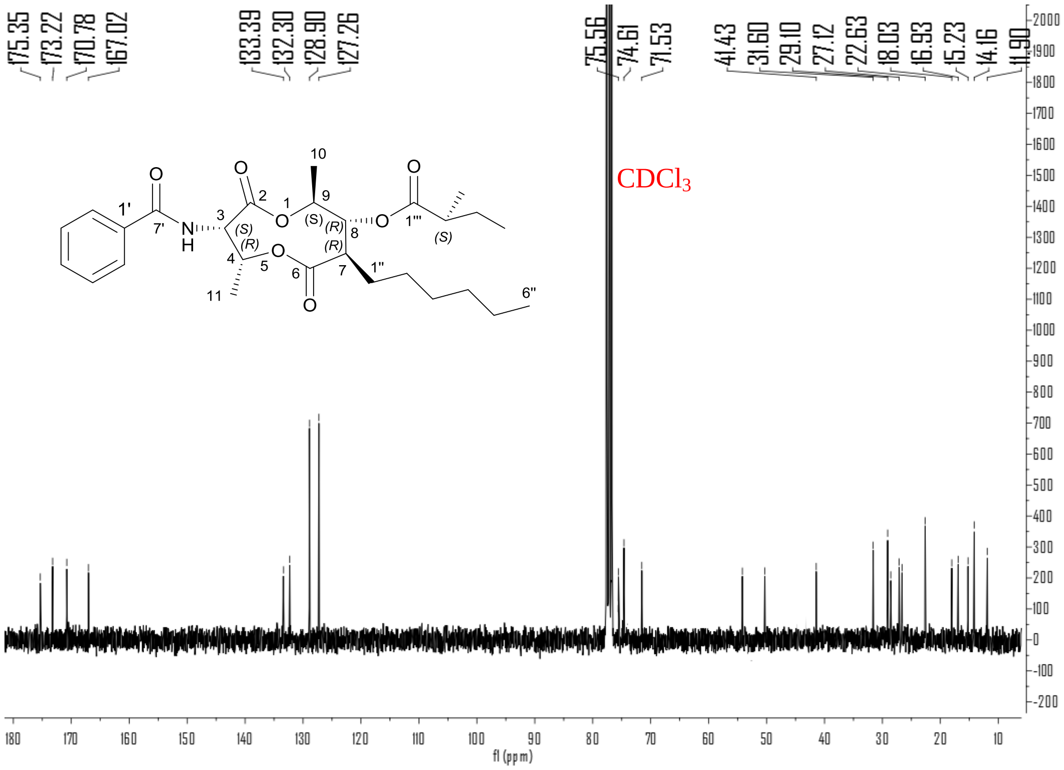

Figure S8. The ^{13}C-NMR spectrum of compound **1**.

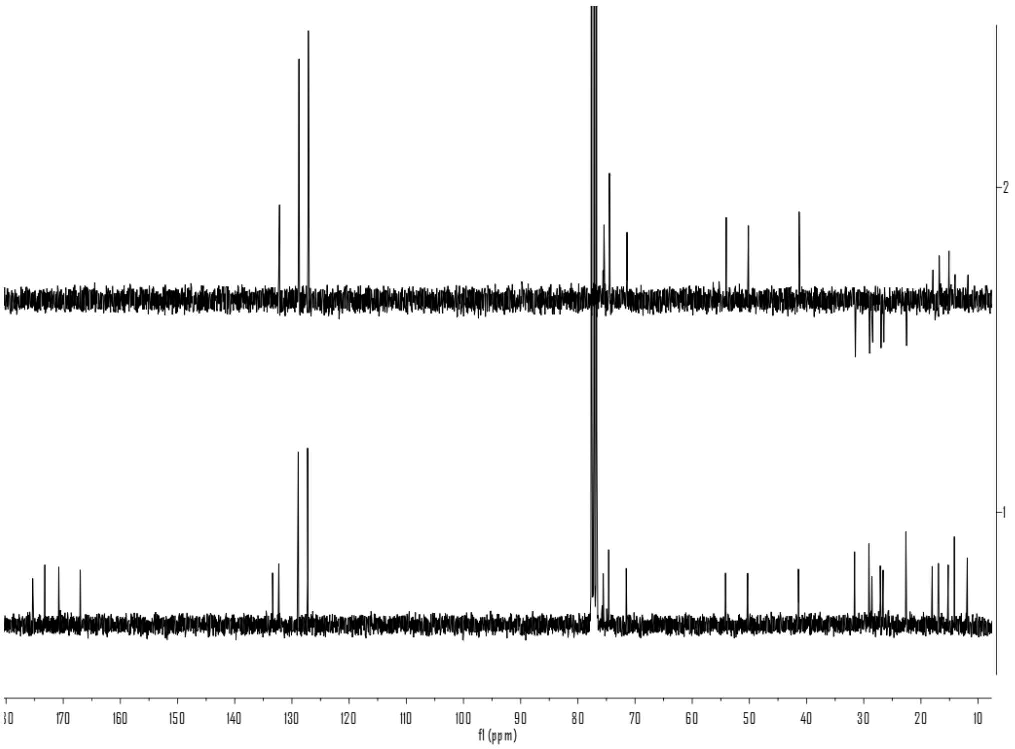

Figure S9. The ^{13}C-NMR and DEPT spectra of compound **1**.

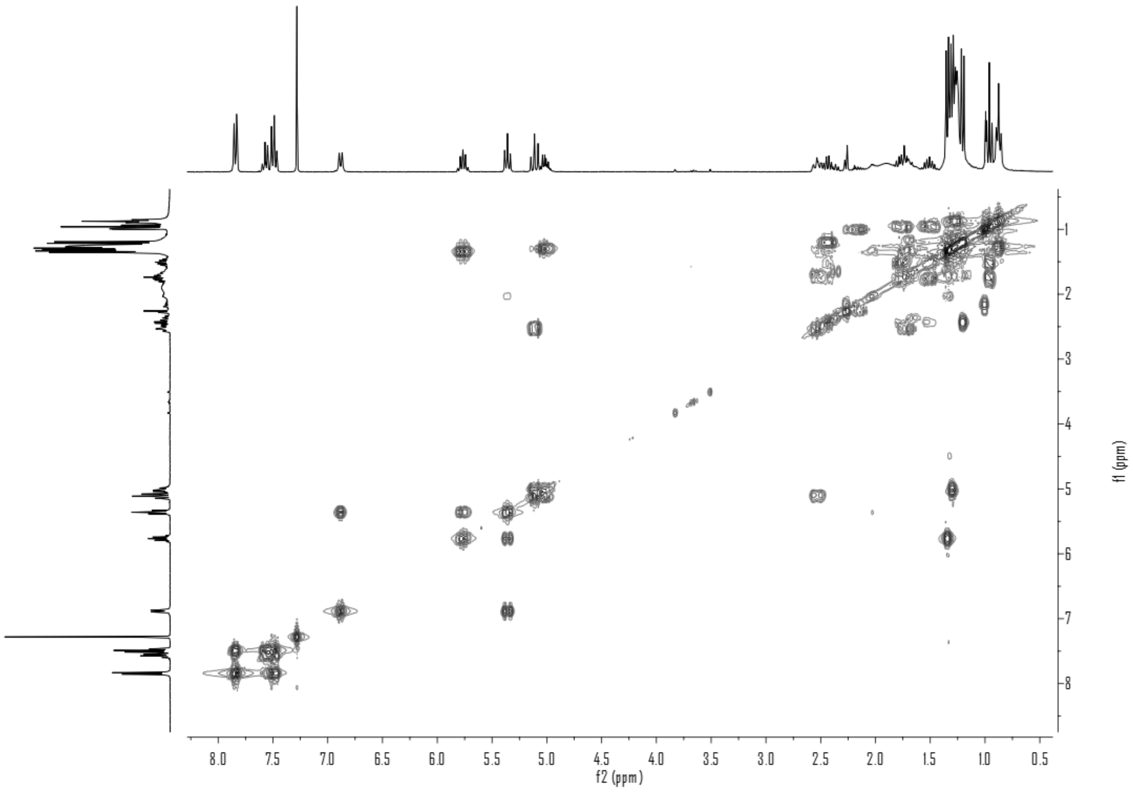

Figure S10. The ^{1}H-^{1}H COSY spectrum of compound **1**.

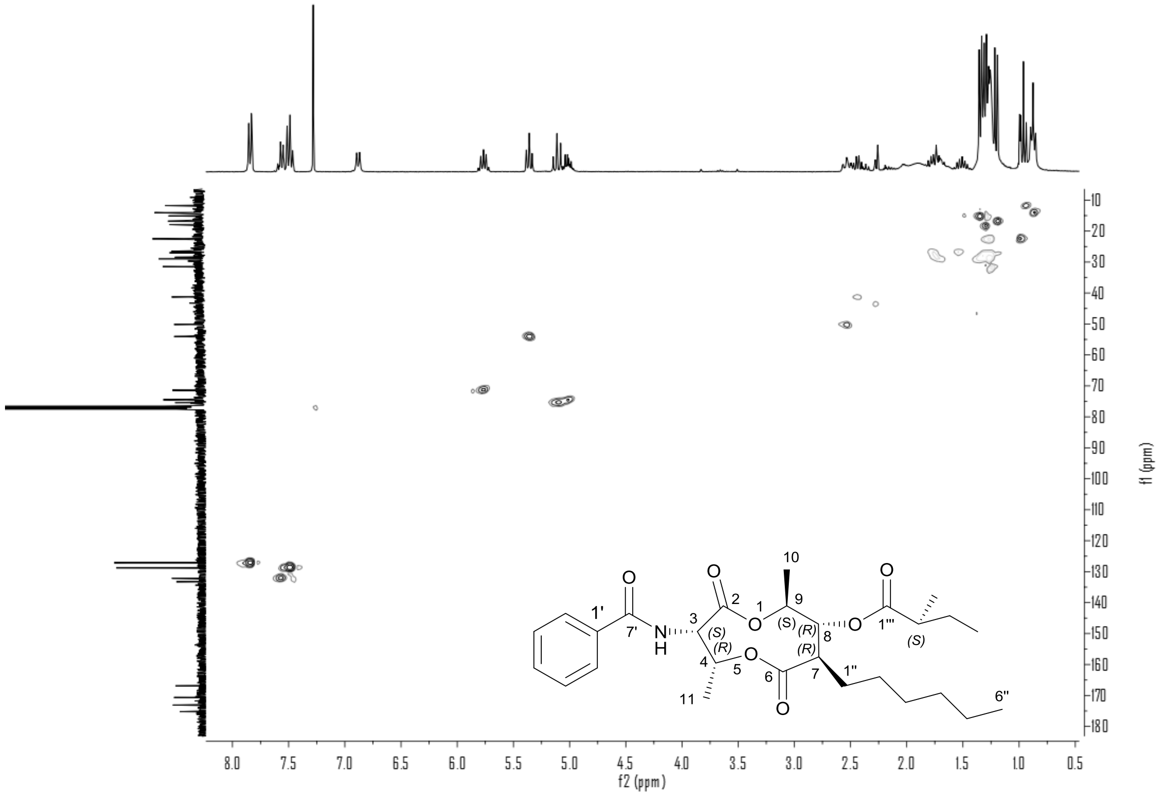

Figure S11. The HSQC spectrum of compound **1**.

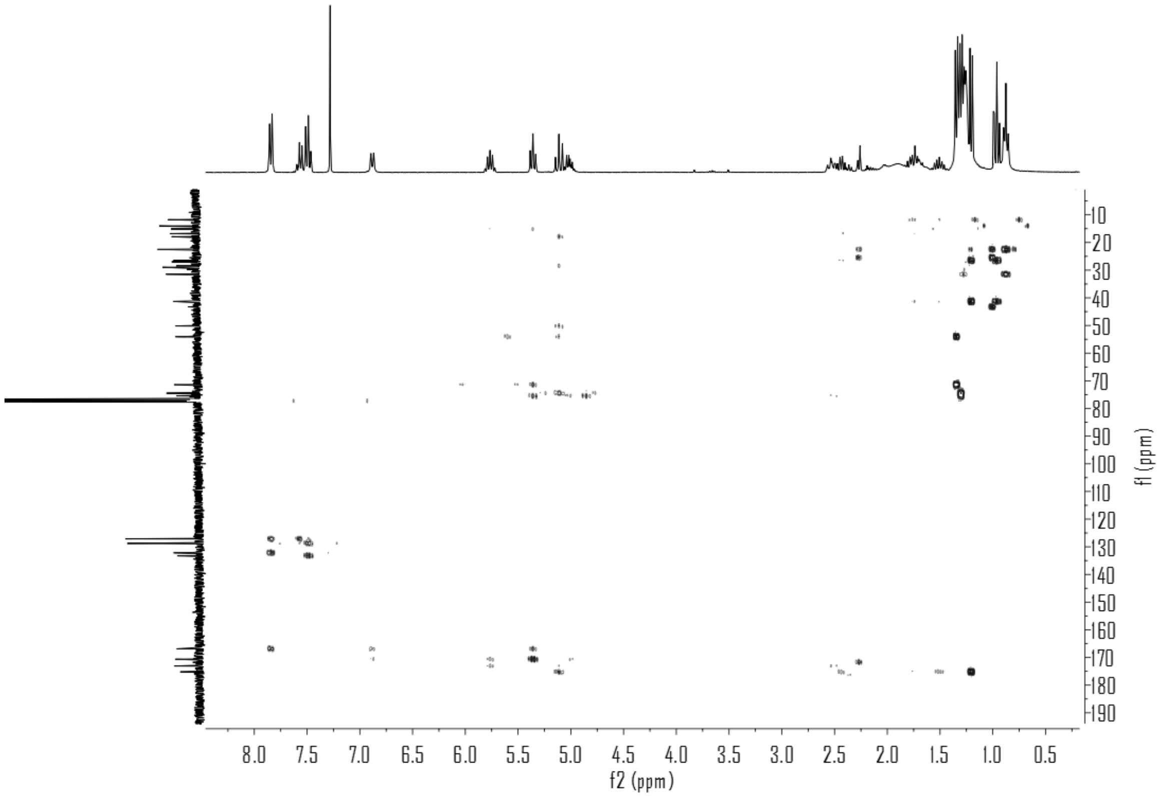

Figure S12. The HMBC spectrum of compound **1.**

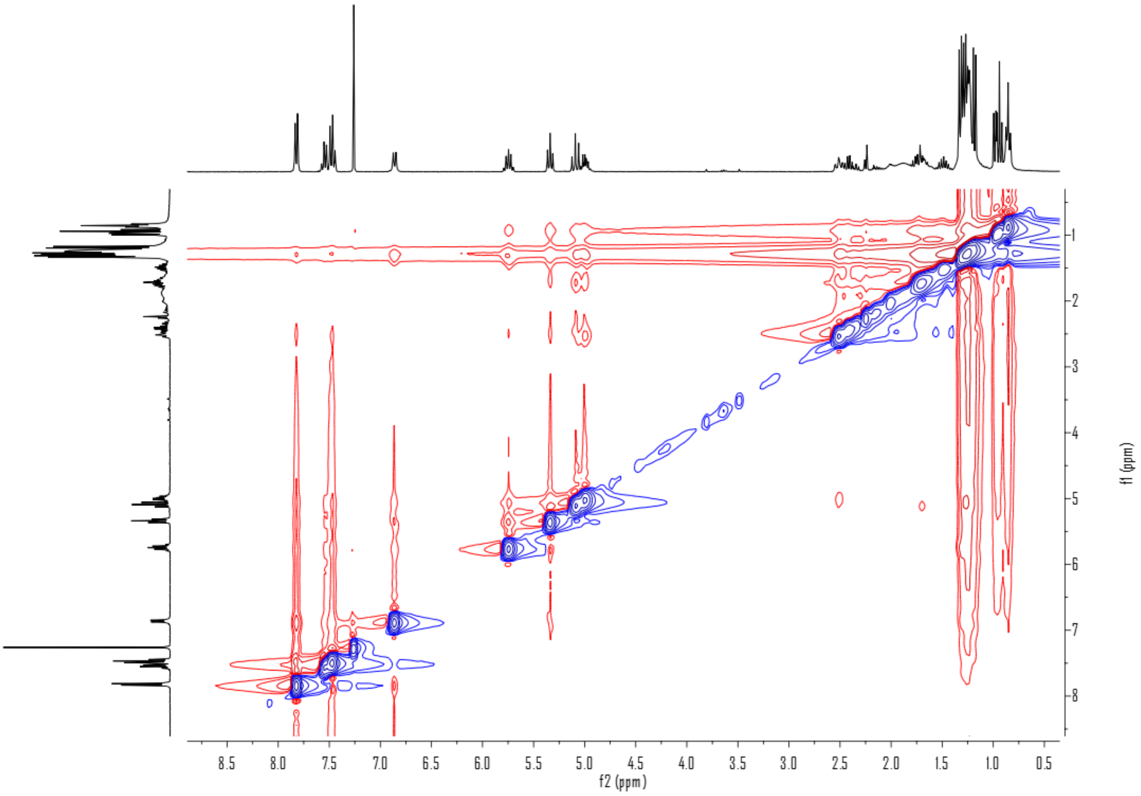

Figure S13. The NOESY spectrum of compound **1.**

The spectra of neoantimycin B (2)

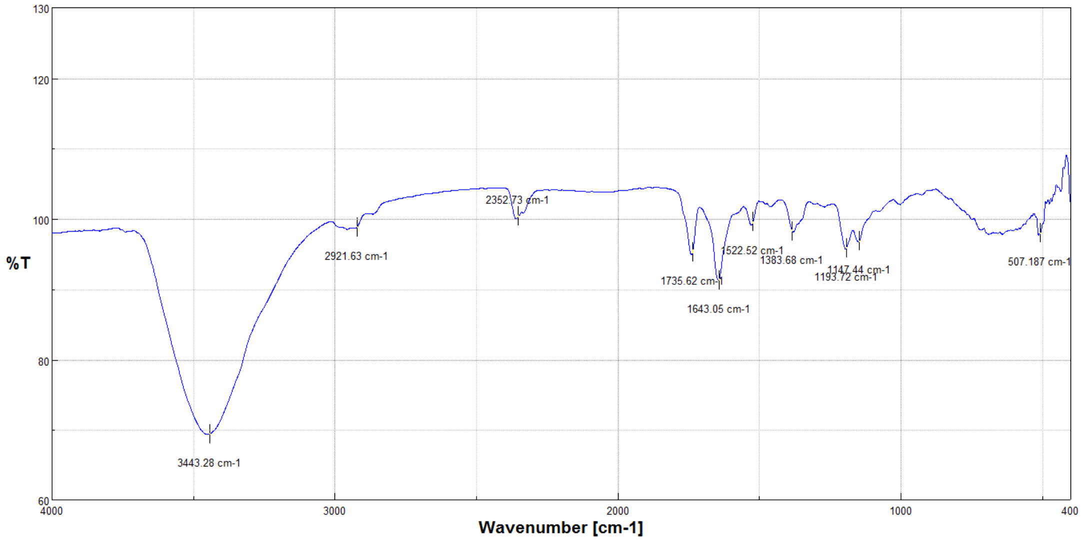

Figure S14. IR spectrum of compound **2.**

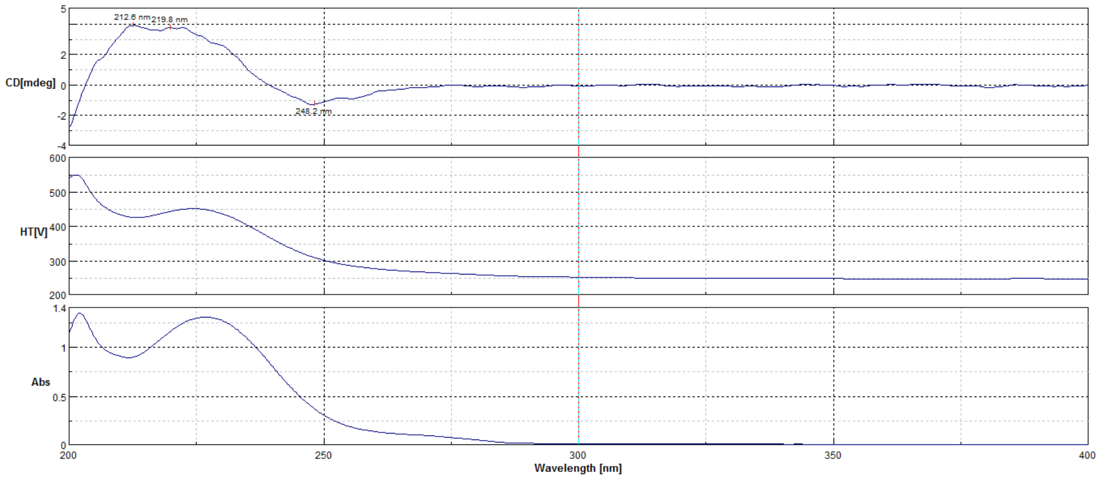

Figure S15. CD spectrum and UV spectrum of compound **2** in MeOH.

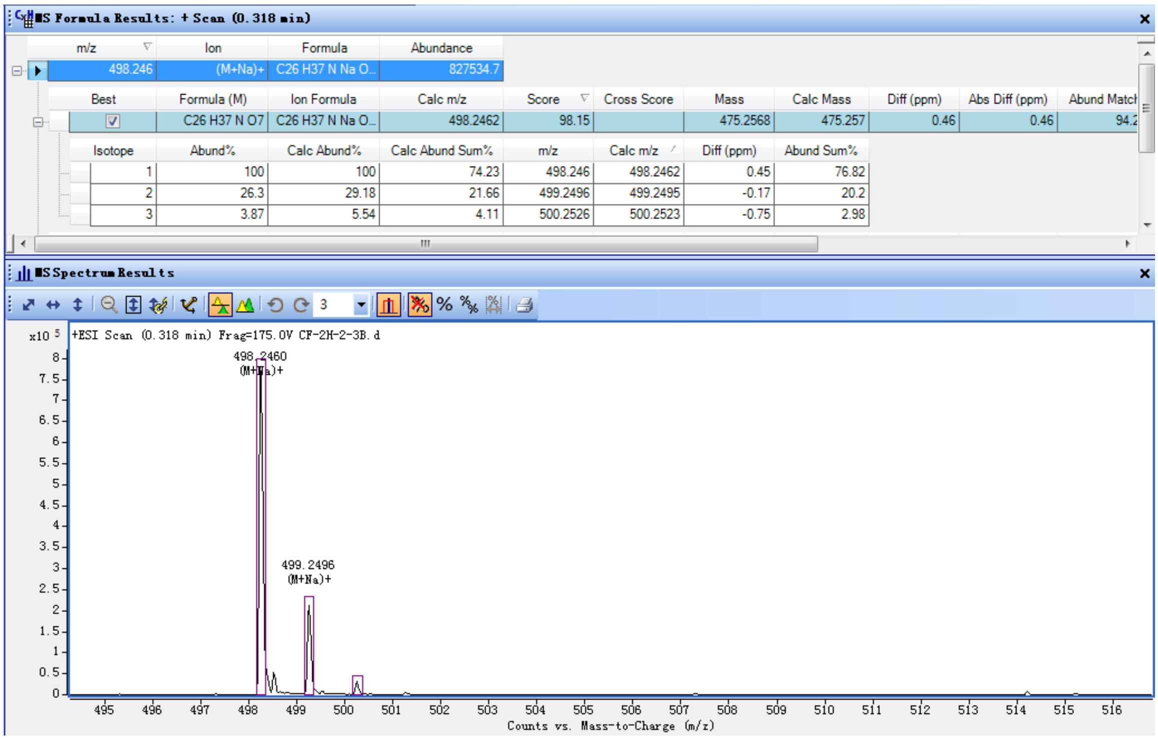

Figure S16. HR-TOF-MS of compound **2**.

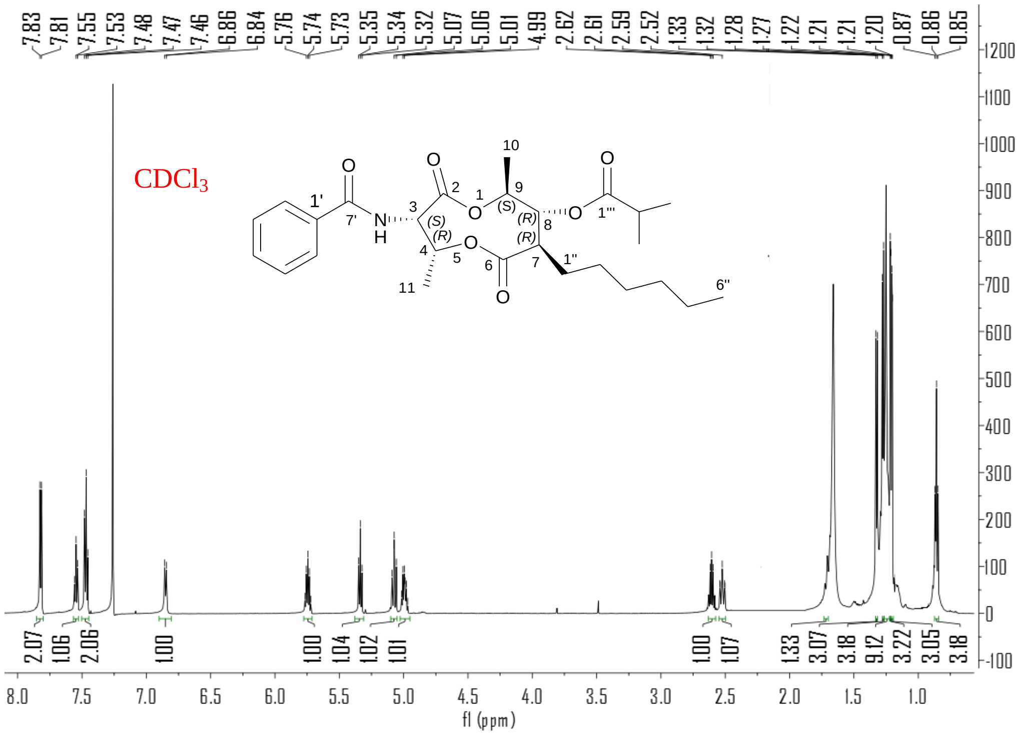

Figure S17. The ^{1}H-NMR spectrum of compound **2**.

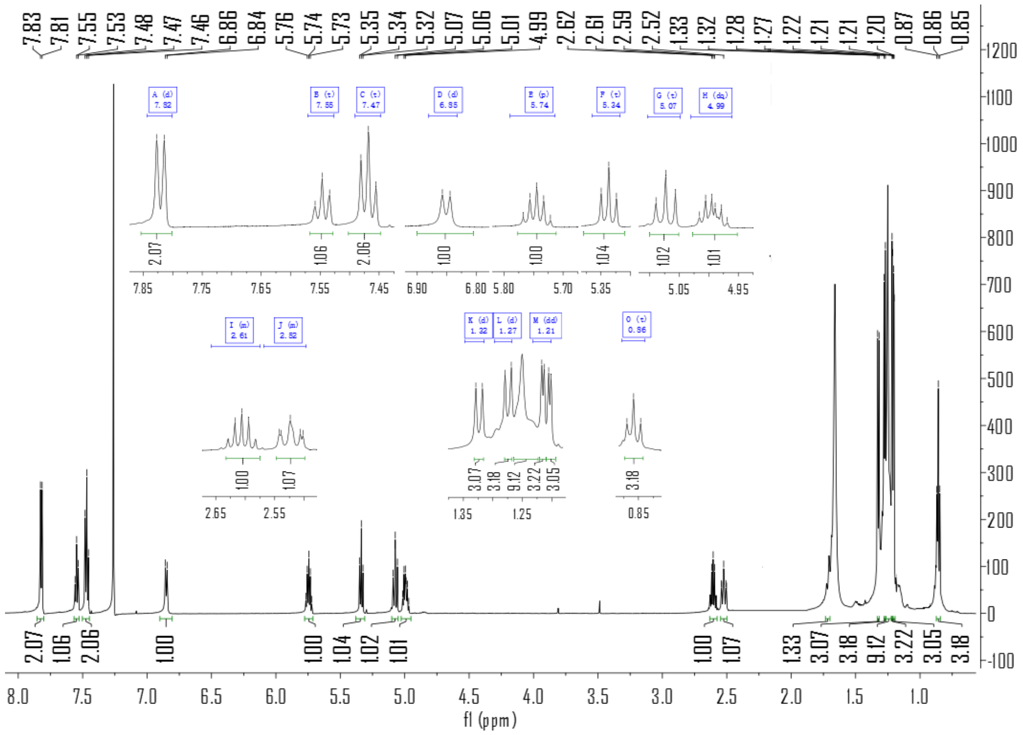

Figure S18. The ^{1}H-NMR spectrum of compound **2.**

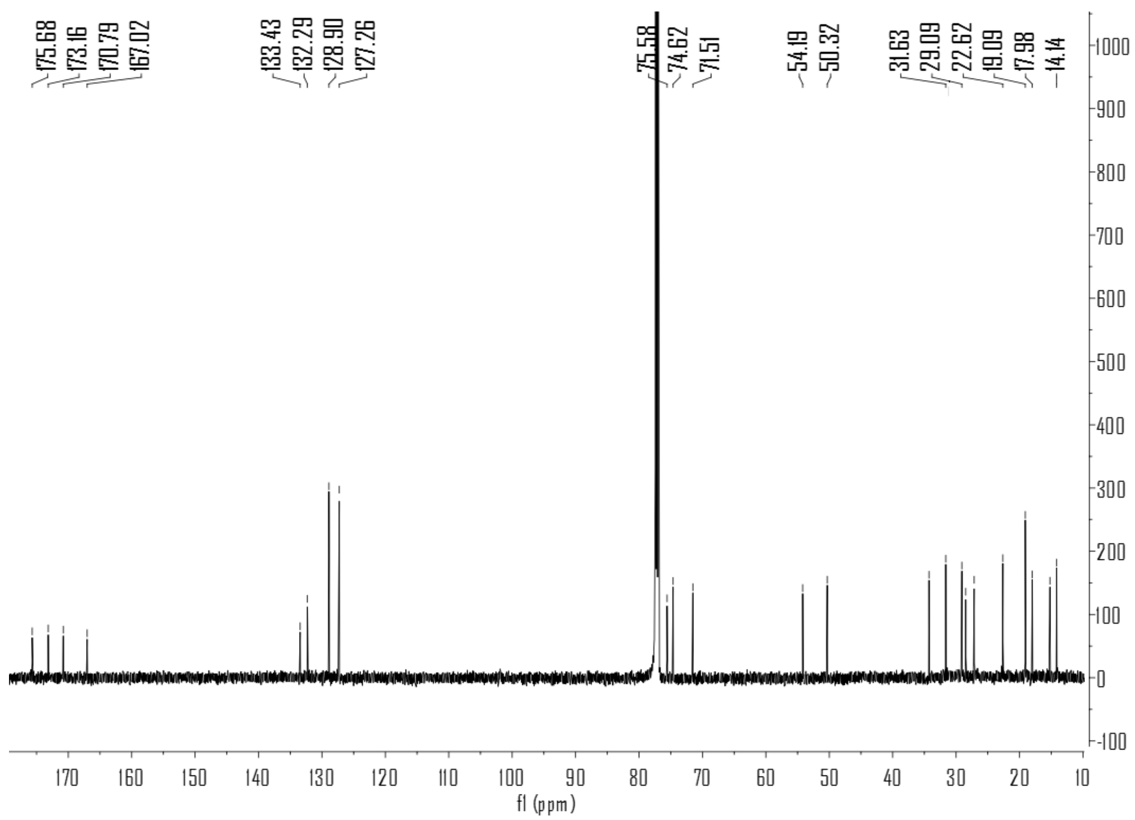

Figure S19. The ^{13}C-NMR spectrum of compound **2**.

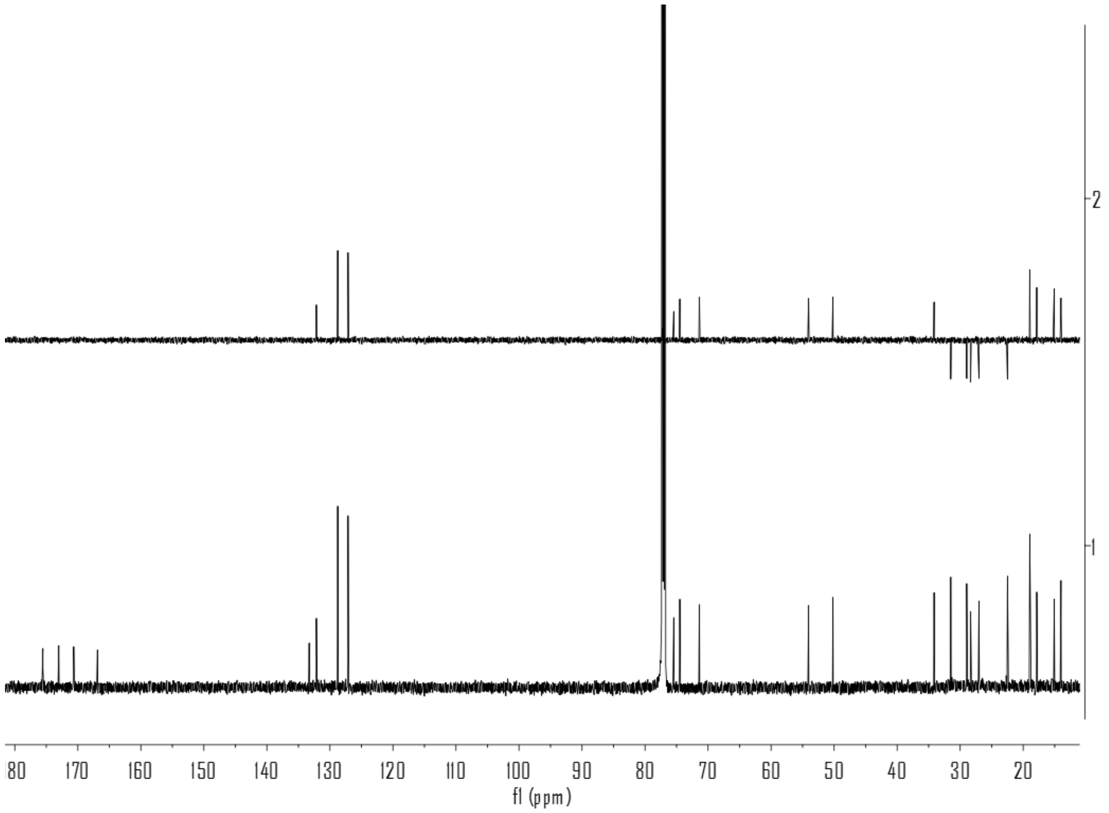

Figure S20. The ^{13}C-NMR and DEPT spectra of compound **2**.

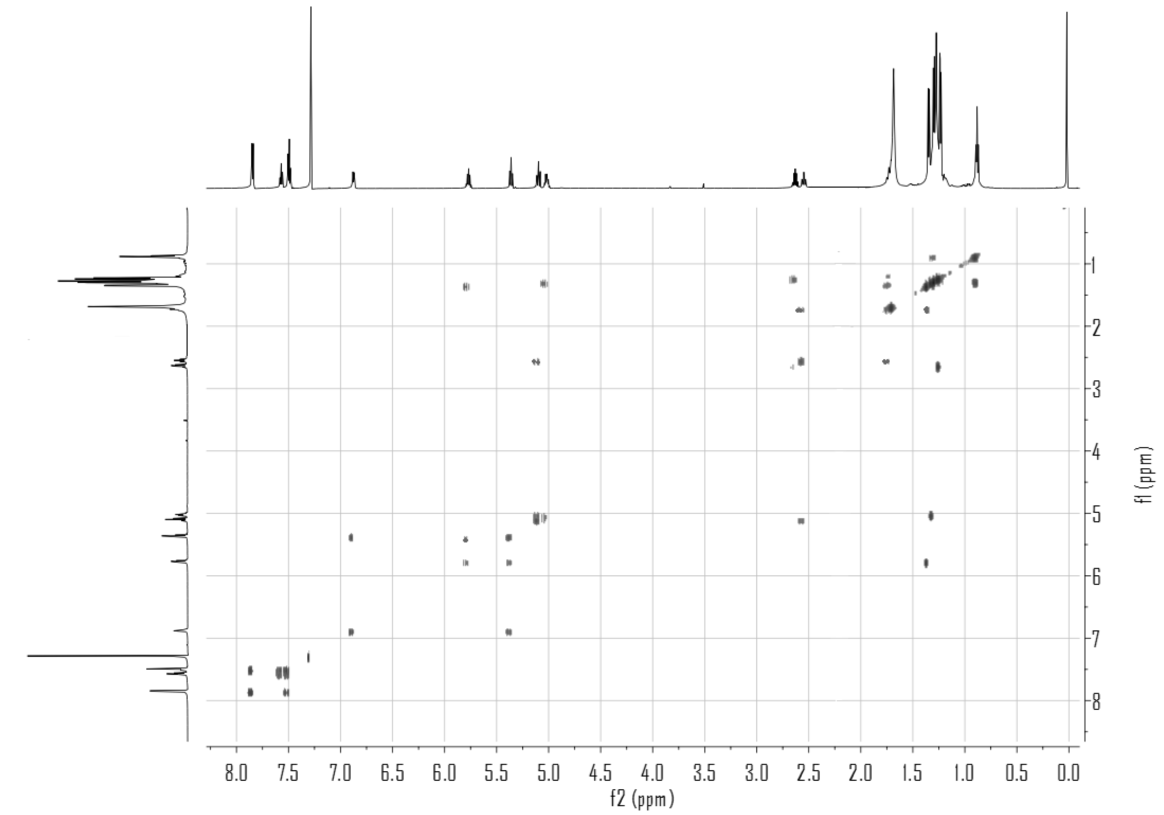

Figure S21. The ^1H-^1H COSY spectrum of compound **2**.

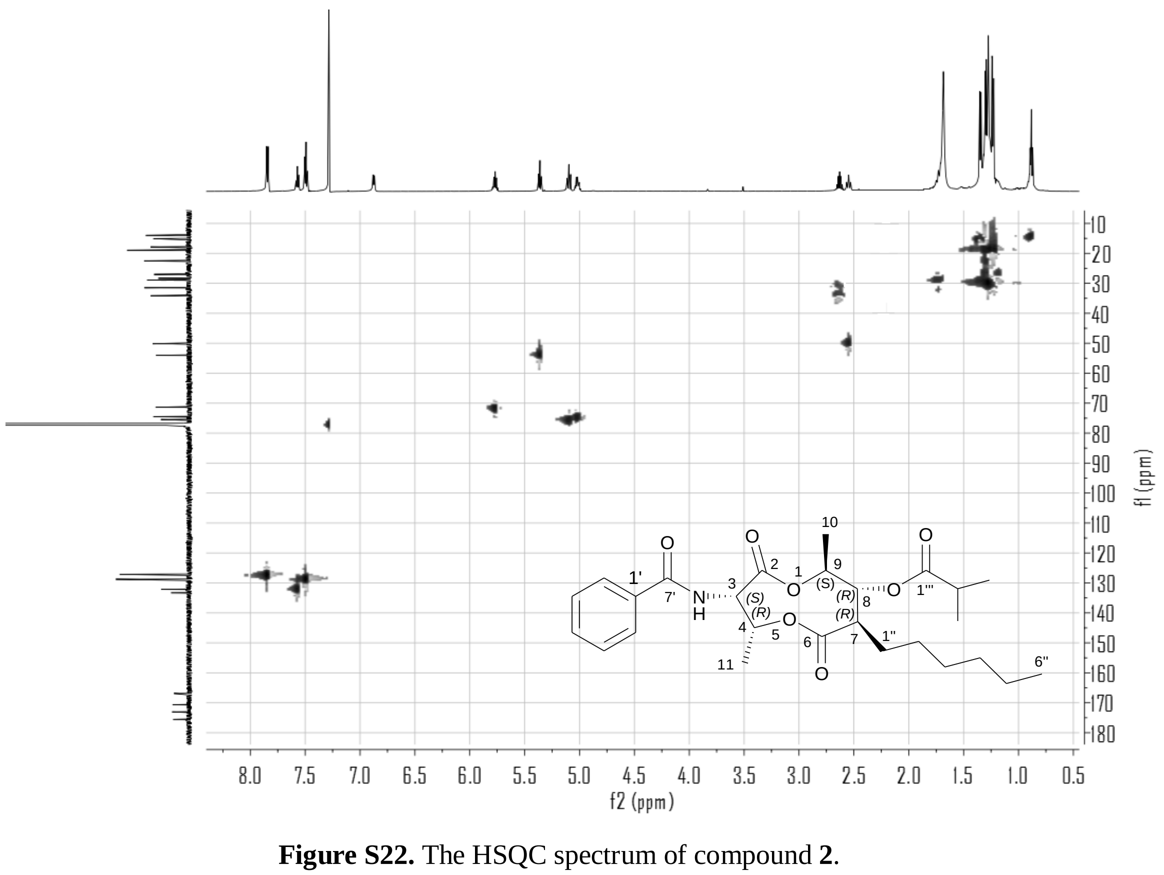

Figure S22. The HSQC spectrum of compound **2**.

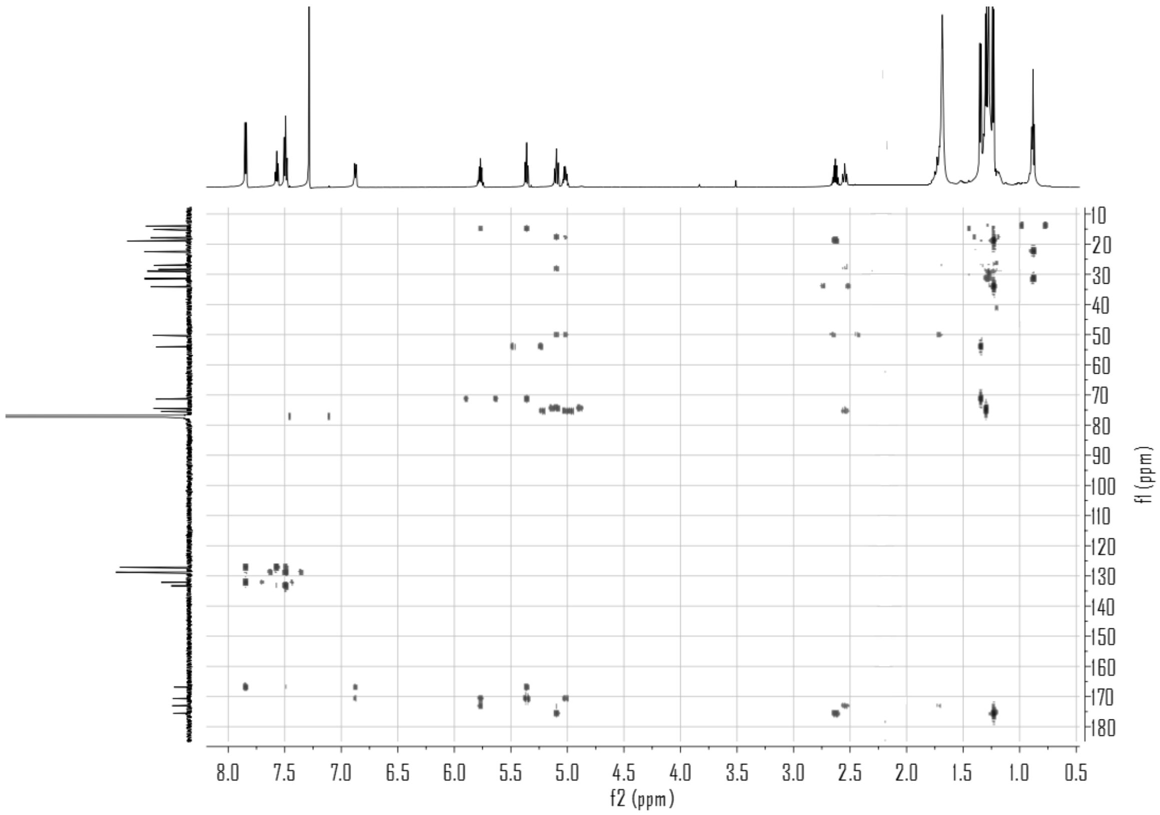

Figure S23. The HMBC spectrum of compound **2**.

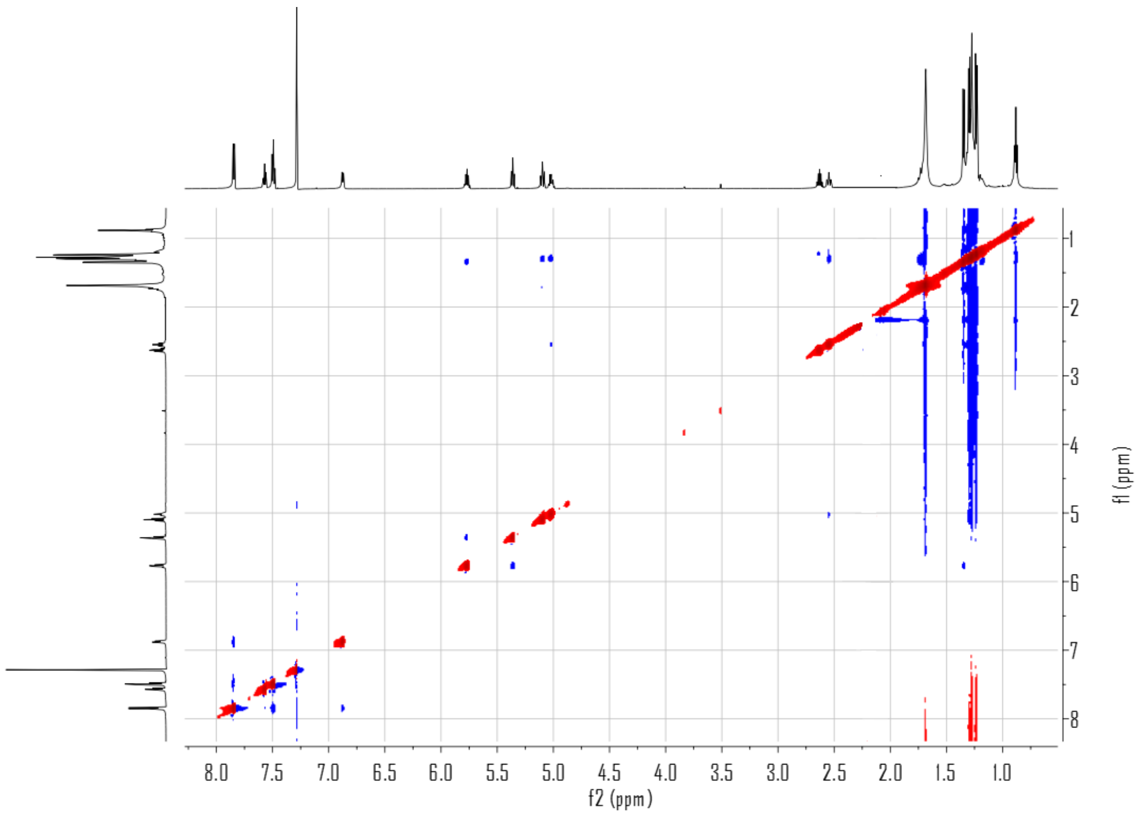

Figure S24. The NOESY spectrum of compound **2**.

Table S5. ^{1}H (300 MHz) and ^{13}C (75 MHz) NMR data of **3-5** (δ in ppm J in Hz).

**No.**	**3**		**4**		**5**	
	δ_C	δ_H	δ_C	δ_H	δ_C	δ_H
**2**	170.1		170.1		170.1	
**3**	53.7	5.32 (1H, t, 7.7)	53.6	5.32 (1H, t, 7.7)	53.6	5.32 (1H, t, 7.7)
**4**	70.9	5.75 (1H, p, 7.0)	70.9	5.75 (1H, p, 7.0)	70.9	5.75 (1H, p,7.0)
**6**	172.9		172.9		172.9	
**7**	50.2	2.52 (1H, m)	50.1	2.52 (1H, m)	50.1	2.52 (1H, m)
**8**	75.4	5.11 (1H, t, 9.9)	75.4	5.11 (1H, t, 9.9)	75.4	5.11 (1H, t, 9.9)
**9**	74.7	4.97 (1H, m)	74.9	4.97 (1H, m)	74.9	4.97 (1H, m)
**10**	17.8	1.28 (3H, d, 6.3)	17.8	1.28 (3H, d, 6.3)	17.8	1.28 (3H, d, 6.3)
**11**	14.9	1.30 (3H, d, 6.7)	15	1.30 (3H, d, 6.7)	15	1.30 (3H, d, 6.7)
**1′**	112.6		112.5		112.5	
**2′**	150.6		150.6		150.6	
**3′**	127.5		127.4		127.4	
**4′**	124.8	8.54 (1H, d, 8.1）	124.8	8.54 (1H, d, 8.1）	124.8	8.54 (1H, d, 8.1）
**5′**	118.9	6.89 (1H, t, 8.1）	119	6.89 (1H, t, 8.1）	119	6.89 (1H, t, 8.1）
**6′**	120.1	7.23 (1H, d, 8.3）	120.1	7.23 (1H, d, 8.3）	120.1	7.23 (1H, d, 8.3）
**7′**	169.4		169.3		169.3	
**8′**	159.1	8.50 (1H, d, 1.7)	159.1	8.50 (1H, d, 1.7)	159.1	8.50 (1H, d, 1.7)
**1″**	28.6	1.68 (1H, m)	28.3	1.68 (1H, m)	28.3	1.68 (1H, m)
**2″**	22.6	1.23 (2H, m)	22.5	1.23 (2H, m)	22.5	1.23 (2H, m)
**3″**	27.2	1.23 (2H, m)	27	1.23 (2H, m)	27	1.23 (2H, m)
**4″**	31.4	1.23 (2H, m)	31.5	1.23 (2H, m)	31.5	1.23 (2H, m)
**5″**	29.2	1.23 (2H, m)	28.9	1.23 (2H, m)	28.9	1.23 (2H, m)
**6″**	14.2	0.85 (3H, t, 7.1)	14	0.85 (3H, t, 7.1)	14	0.85 (3H, t, 7.1)
**7′-NH**		8.43 (1H, d, 8.1)		8.43 (1H, d, 8.1)		8.43 (1H, d, 8.1)
**8′-NH**		9.20 (1H, br.s)		9.20 (1H, br.s)		9.20 (1H, br.s)
**a**						
**1‴**	175.2		175.6		175.6	
**2‴**	41.3	2.41 (1H, m)	34.1	2.61 (1H, t, 7.0)	34.1	2.61 (1H, t, 7.0)
**3‴**	26.5	1.74 (1H, m), 1.50 (1H, m)	18.9	1.22 (3H, d, 2.5)	18.9	1.22 (3H, d, 2.5)
**4‴**	11.7	0.94 (3H, t, 7.4)	18.9	1.21, (3H, d, 2.5)	18.9	1.21, (3H, d, 2.5)
**5‴**	16.7	1.18 (3H, d, 7.0)				
**b**						
**1‴**	171.7					
**2‴**	43.2	2.24 (2H, d, 6.8)				
**3‴**	25.5	2.13 (1H, m)				
**4‴**	22.4	0.98 (3H, d, 6.6)				
**5‴**	22.4	0.98 (3H, d, 6.6)				

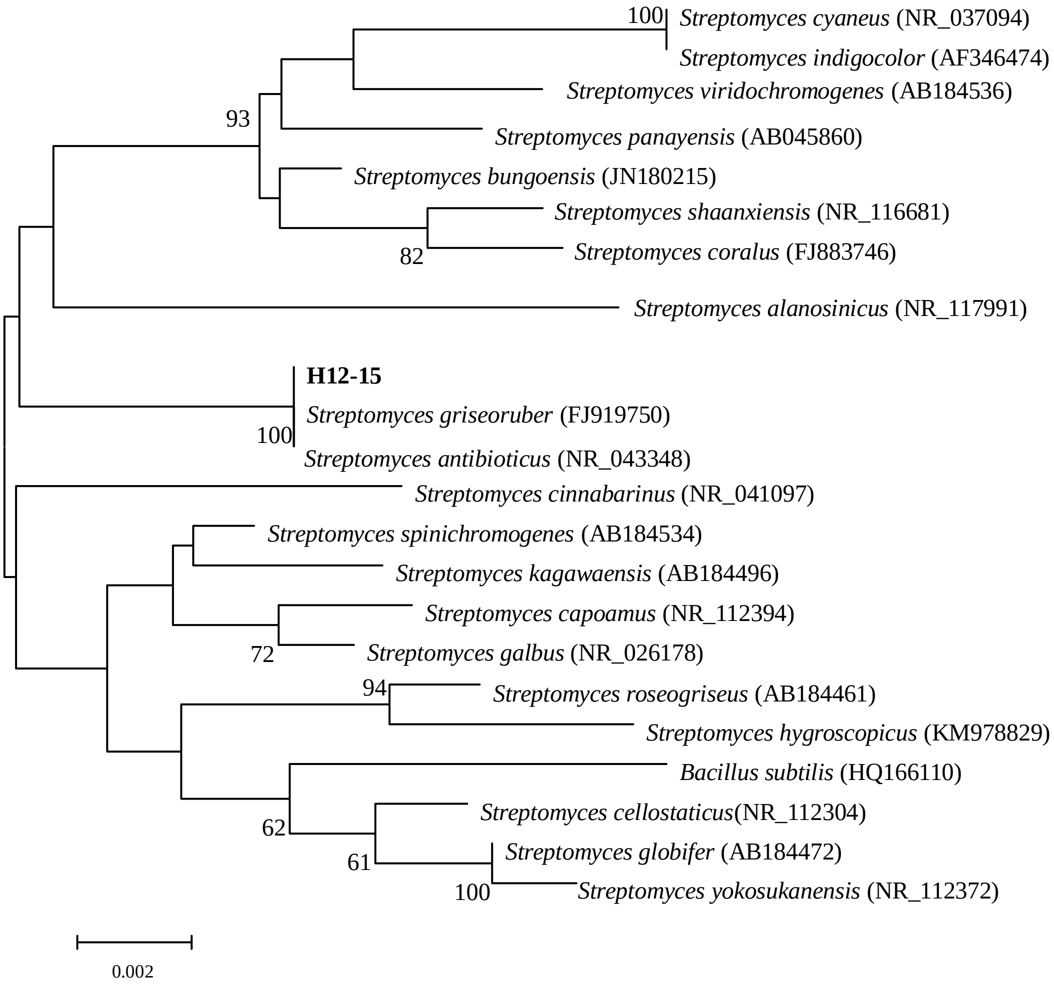

Figure S25. Phylogenetic tree of H12-15.

1CGGCTTGCCGCGTGCTACCATGCAAGTCGAACGATGAAGCCCTTCGGGGTGGATTAG
58TGGCGAACGGGTGAGTAACACGTGGGCAATCTGCCCTGCACTCTGGGACAAGCCCT
114GGAAACGGGGTCTAATACCGGATATCACTCTTGCAGGCATCTGTGAGGGTCGAAAG
170CTCCGGCGGTGCAGGATGAGCCCGCGGCCTATCAGCTTGTTGGTGAGGTAATGGCT
226CACCAAGGCGACGACGGGTAGCCGGCCTGAGAGGGCGACCGGCCACACTGGGAC
280TGAGACACGGCCCAGACTCCTACGGGAGGCAGCAGTGGGGAATATTGCACAATGG
335GCGAAAGCCTGATGCAGCGACGCCGCGTGAGGGATGACGGCCTTCGGGTTGTAAA
390CCTCTTTCAGCAGGGAAGAAGCGAAAGTGACGGTACCTGCAGAAGAAGCGCCGG
444CTAACTACGTGCCAGCAGCCGCGGTAATACGTAGGGCGCAAGCGTTGTCCGGAATT
500ATTGGGCGTAAAGAGCTCGTAGGCGGCTTGTCACGTCGGGTGTGAAAGCCCGGGG
555CTTAACCCCGGGTCTGCATTCGATACGGGCTAGCTAGAGTGTGGTAGGGGAGATCG
621GAATTCCTGGTGTAGCGGTGAAATGCGCAGATATCAGGAGGAACACCGGTGGCGA
676AGGCGGATCTCTGGGCCATTACTGACGCTGAGGAGCGAAAGCGTGGGGAGCGAAC
731AGGATTAGATACCCTGGTAGTCCACGCCGTAAACGGTGGGAACTAGGTGTTGGCGA
787CATTCCACGTCGTCGGTGCCGCAGCTAACGCATTAAGTTCCCCGCCTGGGGAGTAC
843GGCCGCAAGGCTAAAACTCAAAGGAATTGACGGGGGCCCGCACAAGCAGCGGAG

897CATGTGGCTTAATTCGACGCAACGCGAAGAACCTTACCAAGGCTTGACATACACCG
953GAAACGGCCAGAGATGGTCGCCCCCTTGTGGTCGGTGTACAGGTGGTGCATGGCT
1008GTCGTCAGCTCGTGTCGTGAGATGTTGGGTTAAGTCCCGCAACGAGCGCAACCCT
1063TGTTCTGTGTTGCCAGCATGTCCTTCGGGATGATGGGGACTCACAGGAGACCGCC
1118GGGGTCAACTCGGAGGAAGGTGGGGACGACGTCAAGTCATCATGCCCCTTATGTC
1173TTGGGCTGCACACGTGCTACAATGGCCGGTACAAAGAGCAGCGATACCGTGAGGT
1228GGAGCGAATCTCAAAAAGCCGGTCTCAGTTCGGATTGGGGTCTGCAACTCGACCC
1283CATGAAGTCGGAGTTGCTAGTAATCGCAGATCAGCATTGCTGCGGTGAATACGTTC
1349CCGGGCCTTGTACACACCGCCCGTCACGTCACGAAAGTCGGTAACACCCGAAGC
1403CGGTGGCCCAACCCCTTGTGGGAGGGAGCTTCGAAGTGAACCGAACTT

Figure S26. 16S rDNA gene sequence of H12-15.

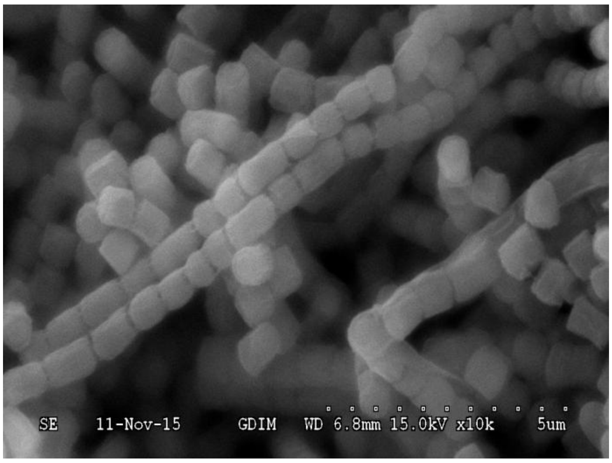

Figure S27. **Scaning electron micrographs of strain H12-15 on Gauze No.1 medium**
